# B Chromosomes Have a Functional Effect on Female Sex Determination in Lake Victoria Cichlid Fishes

**DOI:** 10.1371/journal.pgen.1002203

**Published:** 2011-08-18

**Authors:** Kohta Yoshida, Yohey Terai, Shinji Mizoiri, Mitsuto Aibara, Hidenori Nishihara, Masakatsu Watanabe, Asato Kuroiwa, Hirohisa Hirai, Yuriko Hirai, Yoichi Matsuda, Norihiro Okada

**Affiliations:** 1 Graduate School of Bioscience and Biotechnology, Tokyo Institute of Technology, Yokohama, Japan; 2 Laboratory of Animal Cytogenetics, Faculty of Science, Hokkaido University, Sapporo, Japan; 3 Primate Research Institute, Kyoto University, Inuyama, Japan; 4 Department of Applied Molecular Biosciences, Graduate School of Bioagricultural Sciences, Nagoya University, Nagoya, Japan; Fred Hutchinson Cancer Research Center, United States of America

## Abstract

The endemic cichlid fishes in Lake Victoria are a model system for speciation through adaptive radiation. Although the evolution of the sex-determination system may also play a role in speciation, little is known about the sex-determination system of Lake Victoria cichlids. To understand the evolution of the sex-determination system in these fish, we performed cytogenetic analysis in 11 cichlid species from Lake Victoria. B chromosomes, which are present in addition to standard chromosomes, were found at a high prevalence rate (85%) in these cichlids. In one species, B chromosomes were female-specific. Cross-breeding using females with and without the B chromosomes demonstrated that the presence of the B chromosomes leads to a female-biased sex ratio in this species. Although B chromosomes were believed to be selfish genetic elements with little effect on phenotype and to lack protein-coding genes, the present study provides evidence that B chromosomes have a functional effect on female sex determination. FISH analysis using a BAC clone containing B chromosome DNA suggested that the B chromosomes are derived from sex chromosomes. Determination of the nucleotide sequences of this clone (104.5 kb) revealed the presence of several protein-coding genes in the B chromosome, suggesting that B chromosomes have the potential to contain functional genes. Because some sex chromosomes in amphibians and arthropods are thought to be derived from B chromosomes, the B chromosomes in Lake Victoria cichlids may represent an evolutionary transition toward the generation of sex chromosomes.

## Author Summary

The diversity of sex chromosomes among animal species is well known, but how these sex chromosomes emerged during evolutionary history remains to be solved. One hypothesis for the origin of sex chromosomes is that a portion of the sex chromosome was derived from B chromosomes. In about 10% of eukaryotes, B chromosomes are found in addition to standard chromosomes (sex chromosomes and autosomes). B chromosomes have been thought to be selfish genetic elements with no functional effect on the phenotype of individuals and have been thought to lack protein-coding genes. Although B chromosomes share unique features with sex chromosomes, concrete evidence describing which B chromosomes have evolved to gain a function in sex determination has not been reported. In this study, we found that B chromosomes in one cichlid species from Lake Victoria have a functional effect on sex determination. Moreover, we found that they contained multiple protein-coding genes including morphogenetic related genes. These findings support the hypothesis that a portion of the sex chromosomes has been derived from B chromosomes and shed light on the study of the evolution of sex chromosomes.

## Introduction

The species flock of endemic cichlid fishes in Lake Victoria is the largest known example of recent adaptive radiation and has been highlighted as a model system for the genetic study of speciation [1]–[3]. The evolution of the sex-determination system is suggested to drive the speciation of the cichlids because novel sex-determination genes tend to be associated with novel body colors which can drive reproductive isolation [1], [4]. Recent genetic studies suggest that the sex chromosomes of cichlids have turned over rapidly and that the sex-determination locus is different among species and populations [5]–[7]. Among closely related species of Lake Malawi cichlids, which are sister species of Lake Victoria cichlids, two unlinked sex-determination loci were reported [5]–[6]. In a few populations of Lake Malawi cichlids, multiple interacting loci control sex determination [6], suggesting an ongoing transition in the sex chromosomes. In one species of Lake Victoria cichlids, a sex-determination locus was inferred based on the analysis of a sex-linked phenotype [7]. The sex-determination system has been studied, however, in only a few species of Lake Victoria cichlids. Although cytogenetic analysis is important for studying the sex-determination system, it has not been performed in wild populations in Lake Victoria cichlids. However, a cytogenetic analysis of one species of Lake Victoria cichlid obtained from a commercial source revealed the presence of B chromosomes [8].

B chromosomes are chromosomes found in addition to the standard chromosomes (A chromosomes). They occur in many groups of fungi, plants and animals (10 species, >1300 species, and >500 species, respectively) and vary in number among individuals within a population [9]. They are dispensable for the normal life cycle of host individuals [9]. In most cases, the presence of B chromosomes has no effect on the host phenotype or is deleterious when the number per cell increases [10]. Because of their extensive distribution among many organisms, the possibility that B chromosomes have a beneficial effect on their hosts has been argued [10], but there has been no concrete evidence for this effect. Instead, the existence of B chromosomes has been explained by their selfish behavior such as their non-Mendelian inheritance and accumulation in the germ line [11]. B chromosomes are thought to be composed of repetitive sequences and to lack protein-coding genes [9], and they often form a heterochromatic block [10].

Here we analyzed the karyotypes of wild populations of 11 cichlid species in Lake Victoria. Most individuals possessed B chromosomes of varying sizes. In one species, B chromosomes were specific to females. We performed further analysis of the B chromosomes in this species to reveal their function in sex determination.

## Results/Discussion

### High Prevalence of B Chromosomes in Lake Victoria Cichlids

**Figure 1:**
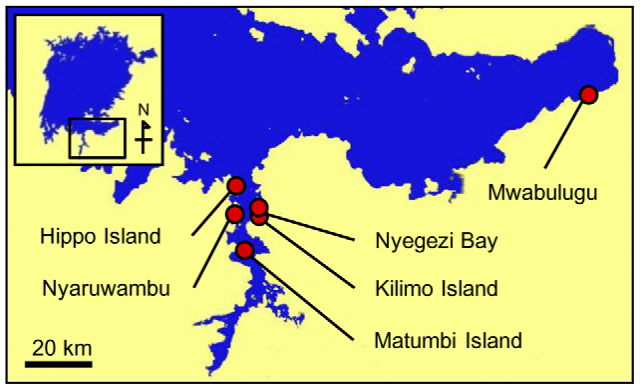
Sampling localities in Lake Victoria. Scale bar, 20 km.

**Figure 2:**
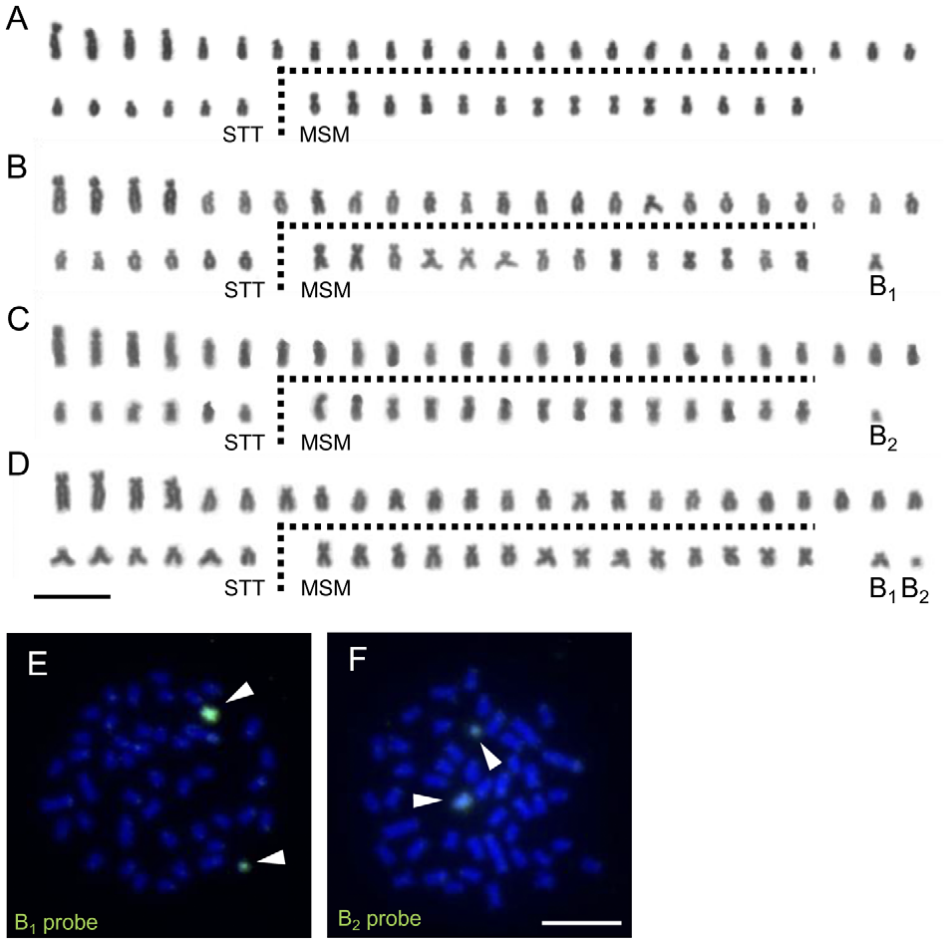
B chromosomes in the pedigree of *L. rubripinnis* from the Matumbi Island population. (A–D) The karyotypes of individuals in one clutch of the pedigree. The larger and smaller B chromosomes are indicated as B_1_ and B_2_, respectively. STT, subtelocentric and telocentric chromosomes; MSM, metacentric and submetacentric chromosomes. (E, F) FISH analysis using B_1_ (E) and B_2_ (F) probes on metaphase spreads from individuals of this pedigree. Arrowheads indicate B_1_ and B_2_ chromosomes. The FISH images were obtained by merging the DAPI-stained patterns (blue) and the signals from the FISH probes (green). Scale bar, 5 µm.

We collected live individuals from six localities (Figure 1) in Lake Victoria during several expeditions in 2005–2007 and 2009 to prepare the chromosome specimens and to produce breeding lines in the laboratory. We analyzed the karyotypes of wild-caught individuals of 11 cichlid species in Lake Victoria (a total of 51 individuals). The chromosome number varied from 44 to 47 and was not specific to the species or populations analyzed (Table 1). To reveal the inheritance pattern of this chromosome number difference, we analyzed the karyotypes of F1 and F2 generation of *Lithochromis rubripinnis* Seehausen, Lippitsch and Bouton, 1998 [12] from the Matumbi Island population (Figure 2A–2D; Table S1). The results indicated that chromosome number varied within clutches by the presence of two small chromosomes, designated as B_1_ (the larger) and B_2_ (the smaller). These data suggested that the chromosome number in the wild-caught individuals varies based on the number of B chromosomes. Their variations in size make it difficult to identify the B chromosomes precisely by morphology.

**Table 1:** Karyotypes of wild-caught individuals of Lake Victoria cichlid species.

Species	Locality	No.	Diploid No.	2*n* = 44	2*n* = 45	2*n* = 46	2*n* = 47
			B No.	B = 0	B = 1	B = 2	B = 3
*Lithocromis rubripinnis*	Matumbi Island	N = 6		2	1	1	2
	Nyaruwambu	N = 2				2	
*Haplochromis plagiodon*	Nyaruwambu	N = 8			6	1	1
*Pundamilia pundamilia*	Nyaruwambu	N = 7			2	3	2
*Haplochromis pyrrhocephalus*	Mwabulugu	N = 6		1	1	3	1
*Neochromis greenwoodi*	Nyaruwambu	N = 5		1	2	2	
*Haplochromis tanaos*	Nyegezi Bay	N = 4		2	2		
*Lithochromis rufus*	Kilimo Island	N = 3			1	2	
	Matumbi Island	N = 1				1	
*Haplochromis* sp. “purple yellow”	Nyaruwambu	N = 3		1	2		
*Neochromis rufocaudalis*	Nyegezi Bay	N = 2			1	1	
	Hippo Island	N = 1				1	
*Haplochromis* sp. “Matumbi hunter”	Nyaruwambu	N = 2			2		
*Haplochromis fisheri*	Nyaruwambu	N = 1				1	
	**Total:**	N = 51		7	20	18	6

**Figure 3:**
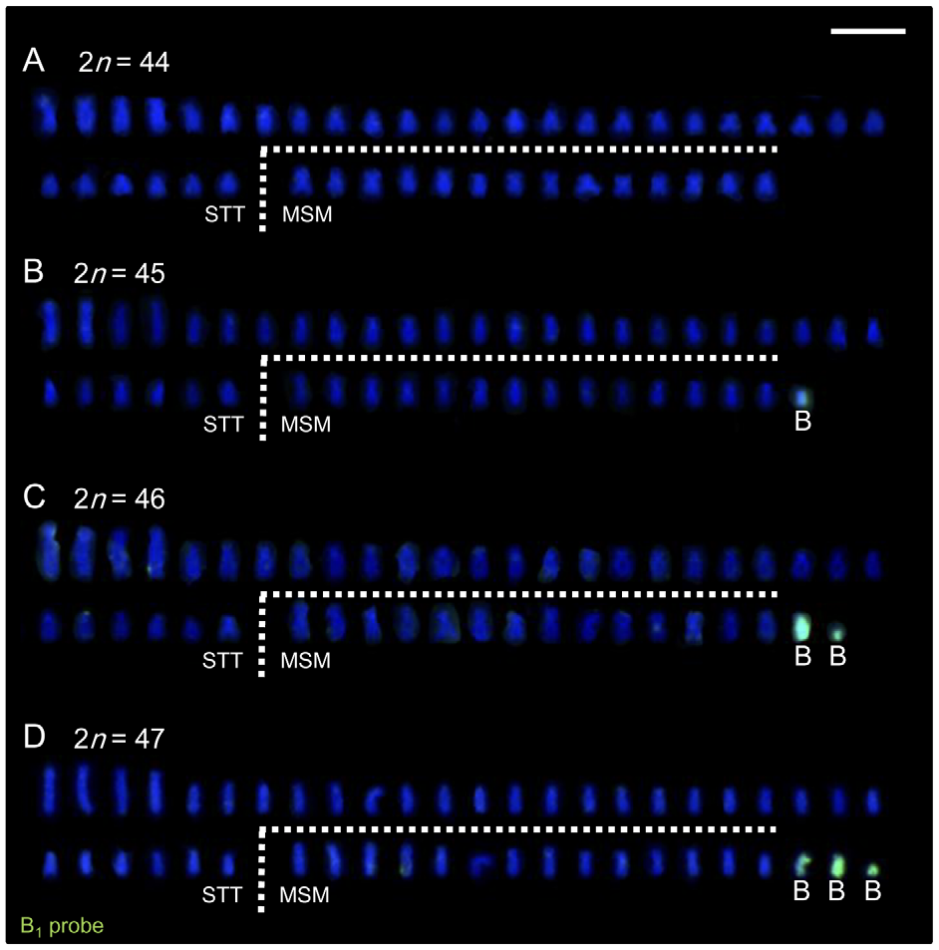
Demonstration of variation in the number of B chromosomes in Lake Victoria cichlids using painting FISH. (A–D) Painting FISH was carried out using the B_1_ probe on karyotypes of wild-caught *L. rubripinnis* individuals that possess 0 (A), 1 (B), 2 (C), and 3 (D) B chromosomes. The images were obtained by merging the DAPI-stained patterns (blue) and FISH signals from the B_1_ probe (green). Scale bar, 5 µm.

B_1_ and B_2_ chromosomes of *L. rubripinnis* were isolated by microdissection and used as probes for FISH analysis. Both of the probes (B_1_ and B_2_ probes) painted both B_1_ and B_2_ chromosomes but not the other chromosomes (Figure 2E and 2F). Although B_1_ chromosomes are larger than B_2_ chromosomes, B_2_ probe painted whole region of B_1_ chromosomes. These results indicated that the B_1_ and B_2_ chromosomes shared repetitive sequences that are specific to B chromosomes. We performed chromosome painting for the wild-caught Lake Victoria cichlids using the B_1_ probes. The number of chromosomes painted varied from zero to three, whereas the number of unpainted chromosomes (44) was the same among all individuals (Figure 3; Figure S1; Table 1; Table S2). These results demonstrate that these cichlids possess 44 A chromosomes and 0–3 B chromosomes. B chromosomes were different in size among populations (Figure S2). The B_1_ probe painted the whole region of all B chromosomes regardless of their size again (Figure S2A), suggesting that B chromosomes are composed of the same B-specific repetitive sequences. B chromosomes were observed in all examined populations at a high prevalence rate (86%; Table 1). The mean number of B chromosomes per individual, however, was low (1.45), suggesting that the accumulation of B chromosomes is restricted. A low prevalence rate of B chromosomes (40%) was reported in breeding individuals of *Haplochromis obliquidens* obtained from the commercial source [8]. This different prevalence rate raises the possibility that B chromosomes may possess some functions that are not essential for the survival of host individuals but are advantageous in wild populations.

### Female-Specific B Chromosomes Have a Functional Effect on Sex Determination

**Figure 4:**
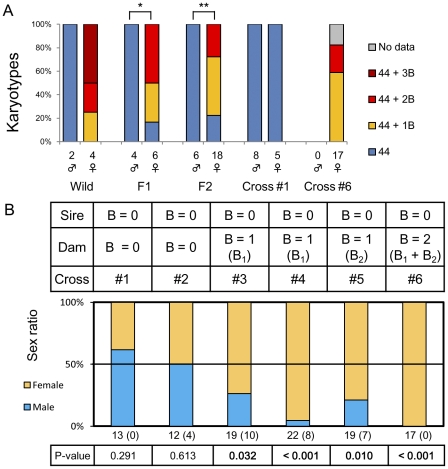
A female-biased sex ratio resulting from the presence of female-specific B chromosomes in *L. rubripinnis*. (A) Correlation between sex and karyotype in the wild-caught individuals and F1 and F2 individuals of *L. rubripinnis* and in offspring from cross-breeding experiments. The number below each histogram indicates the number of individuals. Asterisks indicate the significant association between B chromosomes and females of this species (Fisher's exact test: **P<0.01, *P<0.05). (B) The sex-ratio distortion induced by the presence of the female-specific B chromosome in cross-breeding families. The number of individuals that died as fry is indicated in parentheses. Statistical significance of deviation of sex ratio from 1∶1 was tested by binominal test. P-values are shown in the table below a bar graph.

Although B chromosomes were found in both males and females of most other species (Table S2), in wild individuals of *L. rubripinnis* from the Matumbi island population, all females (N = 4) possessed B chromosomes, whereas males possessed no B chromosomes (N = 2; Figure 4A). We confirmed this female-biased possession of B chromosomes by comparison of karyotypes of males and those of females using F1 and F2 generations in this population. The results showed that almost all females (in a total of N = 24) possessed B chromosomes, whereas no males (in a total of N = 10) possessed B chromosomes (Figure 4A; F1 and F2), indicating that B chromosomes are closely associated with females in this population. We confirmed the absence of B chromosomes in germ cells of F2 males by meiotic analysis (N = 3; Figure S3), indicating that the B chromosomes were specific to females in this population. To reveal the functional effect of B chromosomes on sex determination, we performed cross-breeding experiments between males without B chromosomes and females with different numbers of B chromosomes (0, 1, or 2) and scored the sex ratio of the offspring. The dams without B chromosomes (B^−^) generated nearly 1∶1 offspring sex ratios (proportion of females: 38% in cross #1 and 50% in #2; Figure 4B). These results indicate that one of the sex determination loci is located on an as yet unknown A chromosome. In contrast, the dams with a B chromosome(s) (B^+^) generated female-biased sex ratios. The proportion of females in the offspring was 74%, 91%, 79%, and 100% in cross #3 (B = 1 dam), #4 (B = 1 dam), #5 (B = 1 dam), and #6 (B = 2 dam), respectively (Figure 4B). The correlation of the number of B chromosomes in the dam with the proportion of females in the offspring suggests that the presence of female-specific B chromosomes in this species leads to a female-biased sex ratio. Karyotype analysis of the offspring showed ubiquitous distribution of B chromosomes in offspring from a cross with a skewed sex ratio (cross #6; Figure 4A) and an absence of B chromosomes in offspring from a cross with a nearly 1∶1 sex ratio (cross #1; Figure 4A), confirming the effect of the B chromosomes on sex determination. Although we cannot exclude the possibility that the B chromosomes have a male-specific lethal effect, this is unlikely, because we did not observe a higher death rate in the offspring of the B^+^ dam than in those of the B^−^ dam (Figure 4B, see legend). According to these results, we concluded that B chromosomes have a functional effect on female sex determination. In the offspring of the dam with two B chromosomes (cross #6), four females (29%) possessed two B chromosomes (Figure 4A), indicating non-Mendelian inheritance of the B chromosomes. These observations (i.e., dispensability for host survival and non-Mendelian inheritance) confirmed that these female-specific chromosomes have features that are unique to B chromosomes.

Because all the B chromosomes of several species analyzed here were painted specifically by the B_1_ probe (Figure 3A–3D; Figure S1), they share sequences derived from the same ancestor. Because males in the other populations and species possessed B chromosomes (Table S2), B chromosomes in males have a different effect on sex determination than do the female-specific B chromosomes in spite of their having a shared ancestry. We cannot determine whether the B chromosomes in females in the other populations and species that we examined here are female specific or not. How extensively such female-specific B chromosomes are distributed in cichlids is currently unknown.

The New Zealand frog [13] possesses W chromosomes, which are functionally very similar to the female-specific B chromosomes described above. This species shows a 0W female/00 male sex-determination system. It additionally has B chromosomes that are not female specific and that share partial DNA components with the W chromosome [14]. The W chromosomes are not B chromosomes because they are indispensable for this frog. We speculate that the univalent W chromosome in the frog species might have differentiated from a B chromosome. In most cases, B chromosomes have similar features to sex chromosomes in terms of their meiotic behavior, morphology, and heterochromatic state [15], suggesting the possibility of their evolutionary relatedness. Y chromosomes may also be derived from B chromosomes in a few species of arthropods [16]–[17]. The female-specific B chromosome in cichlids studied here seems to be in an evolutionary transition from B chromosome to sex chromosome. A similar contribution of B chromosomes to the sex ratio in the characid fish *Astyanax scabripinnis* was reported [18], but it is still unclear whether there is a direct connection between the presence of a B chromosome and the sex ratio in those fish. The present case differs from the B chromosome contribution to sex determination found in some arthropods with haplodiploid sex determination because the B chromosome is involved in the exclusion of haploid genomes in those species [19].

### Protein-Coding Genes in the B Chromosomes

**Figure 5:**
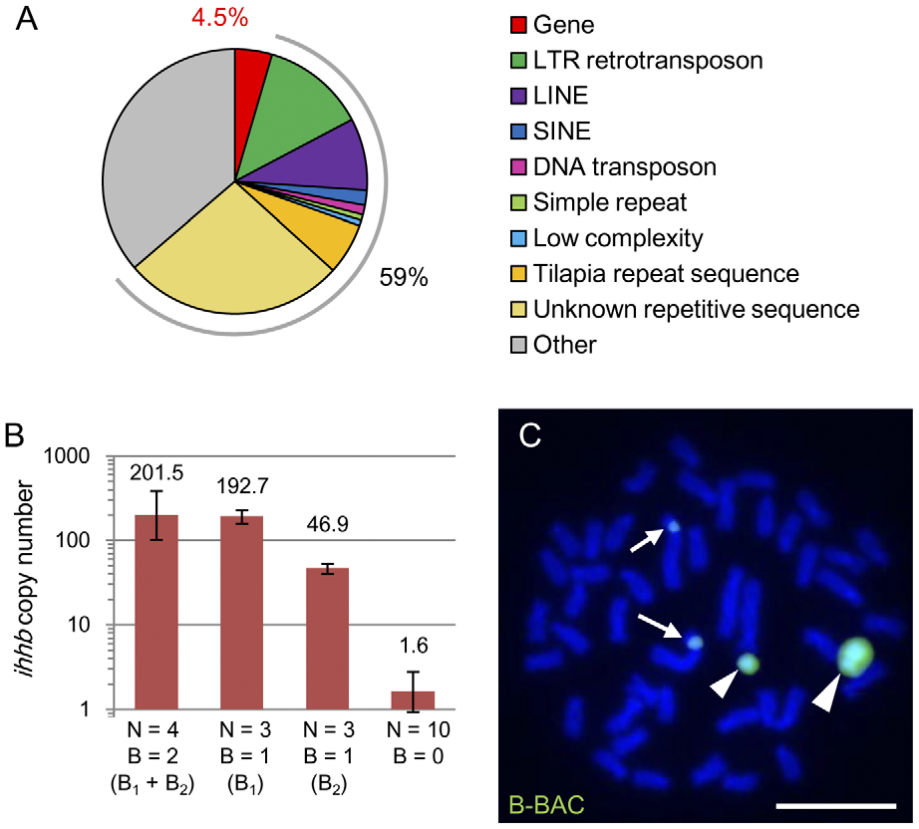
The presence of several protein-coding genes derived from chromosome 1 in B chromosomes. (A) A pie graph showing the composition of the 104.5 kb B-BAC sequence. The percentages of genes and total repetitive sequences are indicated. (B) The copy number of the *ihhb* region as determined by real-time PCR in F2 individuals of *L. rubripinnis* from Matumbi Island population that have different numbers of B chromosomes. The mean value is shown; error bars indicate the standard deviation. (C) FISH analysis using B-BAC as a probe. The images were obtained by merging DAPI-stained patterns (blue) and the signals from the FISH probe (green). Arrowheads indicate B chromosomes. Arrows indicate chromosome 1. Scale bar, 5 µm. Since the B-BAC probe showed stronger signals than the probe using microdissected B chromosome DNA, we detected the homologous autosome region only when we used B-BAC probe.

The functional effect of B chromosomes on sex determination in *L. rubripinnis* suggested the possibility that these chromosomes might have some functional genes. To isolate partial DNA sequences of the B chromosomes, we performed differential screening. We screened B^+^ genomic DNA library and isolated DNA fragments hybridized with a B^+^ genomic probe but not a B^−^ genomic probe. This identified a B chromosome–specific repetitive DNA sequence (named Bseq1; Figure S4). We isolated a BAC clone containing Bseq1 DNA (∼128 kb in total; named B-BAC) from the BAC library constructed from *Haplochromis chilotes* [20]. We analyzed karyotypes of this *H. chilotes* strain (N = 6) and confirmed that all individuals possessed two B chromosomes, indicating strong possibility for inclusion of B chromosome DNA in this BAC library (data not shown). We also confirmed that the BAC clone DNA is derived from B chromosomes by sequence analysis (see below). We determined 80% of the B-BAC sequence (104.5 kb; 18 contigs). Repetitive sequences occupied 59% of the sequence (Figure 5A; Table S3). Remarkably, we discovered five different protein-coding genes in this BAC clone, each of which is almost identical to its parental gene present in the A chromosomes (Table 2; Table S4). The gene density in this B-BAC was higher (4.5%; Figure 5A) than the gene density reported in the cichlid genome (<4%) [21]. No nonsense mutations were found in these genes (1581 a.a. in total). Although protein-coding genes have been reported in B chromosomes in three other cases, i.e. the fungus *Nectria haematococca* [22], several Canidae species [23], [24], and the locust *Locusta migratoria* [25], they have not been thought to have functional significance. The absence of nonsense mutations in the five protein-coding genes in the B chromosomes of cichlids described here might be an indication that the sequence of the B chromosomes has not degenerated from their ancestral sequence, and thus it appears that functional genes have maintained. However, it is possible that their expression might be suppressed by heterochromatin.

**Table 2:** Protein-coding genes in the B-BAC sequence.

Gene (species)	Coding Region	Length (a.a.)	Identity
Indian hedgehog homolog b (*Danio rerio*)	exon 2	87	94%
Lysosomal α-mannosidase (*Danio rerio*)	exons 10–22 (without exons 13, 14)	447	77%
Ribonuclease-like 2 (*Danio rerio*)	whole	140	41%
VPS10 domain receptor protein SORCS 3–like (*Danio rerio*)	exons 1, 2	390	31%
Ryanodine receptor–like unnamed protein (*Tetraodon nigroviridis*)	exons 60–67, 70, 79–82	516	75%

One example of the genes identified in the B chromosome is a morphogenesis-related gene, *indian hedgehog b* (*ihhb*). We estimated the copy number of *ihhb* in the genomes of B^+^ and B^−^ individuals of *L. rubripinnis* by quantitative PCR (Figure 5B). The copy numbers of *ihhb* in the diploid genomes with 2 (B_1_ and B_2_), 1 (B_1_ or B_2_), and 0 B chromosomes, were estimated at 202, 193, 47, and 1.6, respectively. These results showed that there are >40 copies of the *ihhb* paralogs on B chromosomes whereas there is a single copy of the *ihhb* ortholog on the A chromosomes. Sequence analysis provided a tool for distinguishing the paralogs on the B chromosome from the orthologs on the A chromosome. Direct sequencing of *ihhb* exon 2 and the flanking region (Figure S5; total, 2387 bp) using B^+^ and B^−^ genomes showed that *ihhb* orthologs on A chromosomes had a C and T at sites −940 and −88, respectively, in the *ihhb* region, whereas almost all *ihhb* paralogs on B chromosomes had a T and C at the same sites (20 individuals each; Figure S5). These results suggested that the *ihhb* gene was duplicated from an A chromosome to a B chromosome and formed multiple paralogs in B chromosomes. The B-BAC sequence contained the *ihhb* paralog sequence, indicating that the DNA fragment in the BAC clone was indeed derived from a B chromosome. Phylogenetic analysis of the *ihhb* regions (Figure S6) confirmed that these paralogs in the B chromosome emerged from their orthologs in the young Lake Victoria cichlid lineage.

### The Origin and Evolution of B Chromosomes in Lake Victoria Cichlids

FISH analysis using the B-BAC as a probe for *L. rubripinnis* showed intense signals on the short arm of the largest chromosome (chromosome 1) as well as on the B chromosomes (Figure 5C), suggesting that chromosome 1 is a strong candidate for the origin of the B chromosomes. Linkage group 3 (LG3) is a sex chromosome in Tilapia, which is a species that is related to Lake Victoria cichlids [26]. Markers for LG3 (GM385 locus and *dmrt4* (*dmo*) gene) [26] were mapped to chromosome 1 in Lake Victoria cichlids (Figure S7). These findings suggest that the sex chromosome in Tilapia corresponds to chromosome 1 in Lake Victoria cichlids and that the sex-determination-related gene might be located on chromosome 1 of Lake Victoria cichlids. It is likely that the B chromosome in Lake Victoria cichlids has evolved from a part of chromosome 1 that contains the sex-determination-related gene and ultimately gained a function for sex determination in some lineages (the model is presented in Figure S8). However, we could not find genes related to sex determination in the B-BAC sequence. Further study of the sequence of the B chromosome is required to identify the genes that influence sex determination.

In this paper, we showed the recent evolution of a sex-determination system driven by female-specific B chromosomes in Lake Victoria cichlid fishes. The evolution of a sex-determination system can resolve sexual conflict [5], [27], [28]. Sexual conflict can arise when sexually antagonistic genes, which are beneficial to one sex and detrimental to the other, are found on autosomes. However, sexual conflict can be resolved if a gene experiencing sexual antagonism evolves linkage with a sex-determination gene. In this way, the evolution of a new sex-determination locus might resolve sexual conflict [27]. In fact, the sexual conflict produced by the orange-blotched body color pattern, which is beneficial to females but detrimental to males, has been resolved by the emergence of a new sex-determination locus in Lake Malawi cichlids, and the appearance of this color pattern is female-specific [5]. The evolution of this female-specific body color pattern possibly causes sexual isolation by male mate choice of this pattern in cichlids [7]. A direct association between the evolution of a new sex chromosome and sexual isolation was reported in sticklebacks [28]. By linking with the newly emerged female-specific sex determination locus, the female-beneficial sexual antagonistic traits such as female preference to male coloration that have generally observed in Lake Victoria cichlids [3] might have evolved rapidly and have driven speciation. It is, therefore, important for the study of speciation via sexual isolation to analyze the recent evolution of the sex-determination system caused by the female-specific B chromosomes that we have described here in Lake Victoria cichlids. Further studies of the molecular components of B chromosomes as well as the function of B chromosomes in wild populations of cichlids will shed light on the molecular mechanism of how and why a novel sex-determination system emerged during the evolution of these fish.

## Materials and Methods

### Animals

We collected live individuals of 11 species from 6 localities in Lake Victoria (Figure 1) during expeditions in 2005–2007 and 2009. The live fish were shipped to the Tokyo Institute of Technology in Japan for chromosome preparation, extraction of genomic DNA, and cross-breeding. The Malawi cichlid species (*Cyrtocara moorii*, *Fossorochromis rostratus*, *Tyrannochromis macrostoma* and *Petrotilapia tridentiger*) and Tanganyika cichlid species (*Simochromis pleurospilus* and *Perissodus microlepis*) were obtained from traders.

### Cross-Breeding

We crossed females of *Lithochromis rubripinnis* with conspecific males. F1 offspring were allowed to sib-mate to produce the F2 generation. Five F3 families (#1, #3, #4, #5, and #6) were produced by controlled crosses of one F2 male without B chromosomes and five F2 females with zero, one, or two B chromosomes. One F4 family (#2) was produced by a controlled cross of F3 parents without B chromosomes. The sex ratios of these F3 families and the F4 family were scored. Sex ratios were defined as the proportion of males in each clutch and were scored by counting the number of males with breeding coloration as described [29]. All fry exhibited cryptic coloration after hatching, but males begin to display breeding coloration 140 days after their birth [29]. Between 140 to 300 days, all males exhibited breeding color, and sex ratios were scored for each clutch within that time period. We confirmed that sex scored by this method was consistent with the gonadal sex by sacrificing 10 B^−^ males, 10 B^+^ females and 10B^−^ females and observing their gonads.

### Chromosome Preparation and Karyotyping

Chromosome preparation was performed as described [30], [31], with modifications. Chromosomes were prepared from cells of the caudal fin. Caudal fin tissue was cut into small pieces and placed on a collagen-coated dish (IWAKI, Tokyo, Japan). The cells were cultured in Leibovitz's L-15 medium (Invitrogen-GIBCO, Carlsbad, CA) supplemented with 20% fetal bovine serum, 1× antibiotic-antimycotic (PSA; Invitrogen-GIBCO), and 0.1 mg/ml kanamycin sulfate (Meiji Seika, Tokyo, Japan) at 28°C. Non-adherent cells appeared and increased from the caudal fin tissue for 30 days after the initiation of the culture. The cells were harvested after colcemid treatment (0.5 µg/ml) for 2 h, suspended in 0.075 M KCl, fixed three times in 3∶1 methanol/acetic acid, and then dropped onto glass slides and air-dried. More than 30 metaphase spreads for each individual were used for karyotyping. The nomenclature of chromosome morphology as suggested by Levan *et al.* was used [32], providing for two categories with different arm ratios (r): metacentric-submetacentric (MSM, 1<r≤3) and subtelocentric-telocentric (STT, r>3).

### B Chromosome Microdissection and Probe Production

B chromosome microdissection and degenerate oligonucleotide–primed PCR (DOP-PCR) were performed as described [33], [34], with modifications. A single microdissected chromosome fragment, which was sufficient to produce the painting probes, was scraped into a tube. DNA from the scraped chromosome was amplified by first-generation DOP-PCR in a final volume of 15 µl containing 1.5 µl Thermo Sequenase DNA polymerase (GE Healthcare, Chalfont St Giles, UK), 1.5 µl Thermo Sequenase reaction buffer, 0.2 mM dNTPs, and 4 µM primer 6MW (5′-CCGACTCGAGNN NNNNATGTGG-3′). The first-generation DOP-PCR was conducted as follows: 10 min at 95°C; 12 cycles at 94°C for 1 min, 2 min at 30°C, a 6-min transition at 30°C–65°C, and a 3-min extension at 65°C; 30 cycles at 94°C for 1 min, 1 min at 56°C, and 3 min at 72°C; a final extension of 8 min at 72°C. The first-generation DOP-PCR product (3 µl) was used for second-generation DOP-PCR in a final volume of 10 µl. The second-generation DOP-PCR conditions were as follows: 5 min at 95°C; 25 cycles of 94°C for 1 min, 1 min at 56°C, and a 3-min extension at 72°C; a final extension of 8 min. The PCR product of the second-generation DOP-PCR (3 µl) was labeled by the third-generation DOP-PCR in a final volume of 10 µl containing 0.12 nmol/µl digoxigenin-11-dUTP (Roche Diagnostics, Basel, Switzerland). The third-generation DOP-PCR conditions were the same as the second-generation DOP-PCR conditions. The product of the third-generation DOP-PCR was used as the B chromosome probe for painting FISH analysis.

The B chromosome repetitive sequence (Bseq1) was amplified using the primers indicated in Table S5 and subcloned into the pGEM-TA plasmid vector (Promega, Madison, WI) to produce the Bseq1 probe. This clone and the BAC clone were labeled by nick translation with biotin-16-dUTP (Roche Diagnostics).

### FISH

FISH analysis was performed as described [30], [31], with modifications. Hybridization was carried out at 37°C overnight. The slides that had been hybridized with the biotin- or digoxigenin-labeled probe were stained with fluorescein-conjugated avidin (Vector Laboratories, Burlingame, CA) or fluorescein-conjugated anti-digoxigenin (Roche Diagnostics), respectively, and stained with 0.25 µg/ml DAPI. FISH images were observed under a fluorescence microscope (Carl Zeiss, Oberkochen, Germany) using the 1 and 17 filter sets.

### Measurement of Chromosome Size

The actual size of all chromosomes was measured in five metaphase plates in a single individual using Axio Vision (Carl Zeiss). The mean size of the A chromosomes was calculated for each metaphase plate. The ratio of the size of a B chromosome to the mean size of the A chromosomes in the same cell was defined as the relative size of the B chromosome. We averaged the B chromosome sizes across the five metaphase plates. When there was more than one B chromosome in a single cell, we distinguished them by size.

### Meiotic Analysis

We analyzed meiotic chromosomes of three F2 males of *L. rubripinnis* separately. Testes of a single male were nicked and suspended for 90 min in 1% sodium citrate and were fixed for 5 min in 1∶1 ethanol/acetic acid. The testes were placed into 3∶3∶4 ethanol/acetic acid/distilled water to extract the testicular cells. The cells were refixed three times in 1∶1 ethanol/acetic acid and then dropped onto glass slides and air-dried.

### Isolation of the Repetitive Sequence Specific to B Chromosomes

We performed differential screening to isolate the DNA fragments from the B chromosome. We first constructed a B^+^ genomic library. Using the DNeasy kit (QIAGEN, Venlo, Netherlands), B^+^ genomic DNA was extracted from *Lithochromis rufus* (2*n* = 46), which possesses two large B chromosomes. B^+^ genomic DNA was partially digested for 15 s with Sau3AI and subsequently subcloned into the pUC19 plasmid vector. We extracted the plasmid DNA from more than 400 clones and chose the plasmids with DNA inserts >500 bp (64 clones).

We separated the chosen plasmid DNAs by 1.5% agarose gel electrophoresis. DNA fragments were transferred from the gels to GeneScreen Plus membranes (Perkin-Elmer, Norwalk, CT) in 0.4 M NaOH and 0.6 M NaCl. Membranes were neutralized in 0.5 M Tris-HC1 (pH 7.0) and 1 M NaCl and then dried. We performed electrophoresis and transfer of the DNA fragments twice using the same amount of DNAs for each plasmid to make two copies of the membranes.

Next, we produced the probe of B^+^ genomic DNA and B^−^ genomic DNA. B^−^ genomic DNA was extracted from *L. rubripinnis* (2*n* = 44, without B chromosome). B^+^ genomic DNA for the probe was extracted from the same individual as that for the genomic library. One microgram of both B^+^ and B^−^ genomic DNA was labeled for probes with [α-^32^P]dCTP using the *Bca*BEST™ labeling kit (Takara, Tokyo, Japan).

Hybridization was performed at 42°C overnight in a solution of 50% (v/v) formamide, 1 M NaCl, 1% SDS, 2× Denhardt's solution, and 100 pg/ml of labeled probe DNA. We used B^+^ and B^−^ labeled probes for each membrane. After the hybridization, we washed the membranes and detected the signals. We compared the signal intensity of the membranes hybridized with the B^+^ probe and with the B^−^ probe. Two clones, including the Bseq1 clone, showed stronger signals in the hybridization with B^+^ than with B^−^. We determined the sequences of these clones. The primers were designed according to the sequence of the Bseq1 clone (Bseq1F, Bseq1R; Table S5). This region was amplified by PCR using B^+^ genomic DNA.

### Sequence Analysis of the BAC Clone

A BAC clone (B-BAC) containing Bseq1 was screened and isolated from the *Haplochromis chilotes* BAC library [20]. B-BAC DNA was digested with BglII, BamHI, HindIII, PstI, XbaI, and SphI. Each of the resultant DNA fragments was subcloned into pUC19. We determined the sequences of the DNA fragments inserted into the plasmid. The flanking sequences were determined by direct sequencing using BAC clone DNA. Repeat masking was performed with RepeatMasker ver. open-3.2.9, with a Teleostei repeat library of database ver. RM-20090604 and the –s (slow and most sensitive) option. Subsequent repeat masking was performed under the same conditions using a tilapia repeat sequence library [35], which contains insufficiently characterized repeat sequences. The sequences in which the repetitive sequences were masked were used for a subsequent NCBI BLAST search (http://blast.ncbi.nlm.nih.gov/Blast.cgi) of the whole-genome shotgun data of five Lake Malawi cichlids (*Maylandia zebra*, *Mchenga conophoros*, *Melanochromis auratus*, *Labeotropheus fuelleborni*, and *Rhamphochromis esox; 21*) to identify unknown repetitive elements, because a number of uncharacterized repeats in the B-BAC sequences from *H. chilotes* were not masked with the RepeatMasker. The partial B-BAC regions that hit at least four different loci in one Lake Malawi cichlid with an E-value of <10^−4^ over 35 nt were chosen as unknown repetitive sequences. The sequences in which both known and unknown repetitive sequences were masked were used for a subsequent NCBI BLASTX search in the non-redundant protein sequence database of bony fishes with an E-value cutoff of 10^−10^. To analyze the coding regions of the five protein-coding sequences precisely, we performed a homology search with them in translated B-BAC sequences using Genetyx ver. 6.1.0 (Genetyx, Tokyo, Japan).

### Sequence Analysis of *ihhb*

The primers for the *ihhb* region are indicated in Table S5 and Figure S5. Two fragments of the *ihhb* region were defined by the position of the primer pair Bseq1F and ihhbR5 and the pair ihhbF3 and ihhbR8. Each fragment was amplified from the genomic DNA by PCR. We purified the PCR products and determined the sequences using the primers Bseq1F, Bseq1R, ihhbF1, ihhbF2, ihhbF4, ihhbF5, ihhbF6, ihhbR1, ihhbR2, ihhbR3, ihhbR4, ihhbR5, ihhbR7, and ihhbR8. When the sequences included heterogeneous sites, we subcloned the PCR products into the pGEM-TA plasmid vector and determined the sequences of several clones to obtain the sequence information and eliminate PCR errors. Phylogenetic analysis was performed using MEGA4.0 [36]. Phylogenetic trees were obtained by neighbor-joining (NJ), minimum-evolution (ME), and maximum-parsimony (MP) methods with bootstrap tests.

### Quantitative Real-Time PCR (qPCR)

The partial fragment of the *ihhb* region was amplified by PCR using the primers ihhbF3 and ihhbR7 and was cloned into the pGEM-TA plasmid vector for the standard control for calibration of qPCR. qPCR was performed in a 12.5-µl reaction using the Quantitative SYBR Green RT-PCR kit (Applied Biosystems, Foster City, CA). PCR amplification and product detection were conducted using Thermal Cycler Dice (TaKaRa) and the primers ihhbF4 and ihhbR6, which were derived from intron 1 and exon 2 of *ihhb*, respectively (Figure S5). The sequences of the primers completely matched the primer-annealing sites in all analyzed genomes. The Ct values were calculated by the second-derivative-maximum method. Relative quantification of the samples was calculated by fitting the Ct value to the standard curve of the vector. The copy number for the genomic DNA was calculated using the concentration and length (3508 bp) of the standard plasmid, together with the genomic size of 1.17×10^9^ bp calculated by the reported C-value (1.2 pg) of the Lake Victoria species *Haplochromis parvidens* [37].

### Accession Numbers

The GenBank (http://www.ncbi.nlm.nih.gov/Genbank) accession numbers for DNA sequences discussed in this paper are: AB601473–AB601502.

## Supporting Information

Figure S1 Painting FISH using the B_1_ probe in the metaphase spread of wild-caught individuals of 10 species of Lake Victoria cichlids. (A) *Haplochromis plagiodon*, (B) *Pundamilia pundamilia*, (C) *Haplochromis pyrrhocephalus*, (D) *Neochromis greenwoodi*, (E) *Haplochromis tanaos*, (F) *Lithochromis rufus*, (G) *Haplochromis* sp. “purple yellow”, (H) *Neochromis rufocaudalis*, (I) *Haplochromis* sp. “Matumbi hunter”, and (J) *Haplochromis fisheri*. The images were obtained by merging the DAPI-stained patterns (blue) and FISH signals from the probe (green). Arrowheads indicate B chromosomes. Scale bars, 5 µm.
(TIF)

Figure S2 Differences in the size distribution of B chromosomes among populations of Lake Victoria cichlids. (A) Size variation of the B chromosomes painted by the B_1_ probe (above) and stained with DAPI (bottom). The size of the B chromosomes was calculated by comparison with the mean size of A chromosomes (indicated as 1.00). The images of B chromosomes were adjusted based on the calculated size. B chromosome sizes (species name) of the images are 1.38 (*Haplochromis plagiodon*), 1.25 (*Lithochromis rufus*), 1.09 (*H. plagiodon*), 0.91 (*H. plagiodon*), 0.79 (*L. rubripinnis*), 0.70 (*L. rubripinnis*), 0.51 (*Pundamilia pundamilia*), 0.40 (*L. rubripinnis*) and 0.24 (*Haplochromis pyrrhocephalus*). (B) The differences in the distribution of the relative sizes of B chromosomes. The ratio of the size of a B chromosome to the mean size of the A chromosomes in the same cell was defined as the relative size of the B chromosome. Mean relative size and standard deviation are indicated above each histogram (N = number of B chromosomes). Populations in which more than four B chromosomes were observed were compared.
(TIF)

Figure S3 Meiotic chromosomes in the males of the pedigree of *L. rubripinnis* from the Matumbi island population. Meiotic chromosomes of the three F2 males of *L. rubripinnis* are indicated in intact (A, C and E) and annotated images (B, D and F). Each chromosome is surrounded by line (B, D and F). The observed haploid number is indicated (*n*). SC, sperm cells. SG, spermatogonia. Scale bar, 1 µm.
(TIF)

Figure S4 FISH analysis using the Bseq1 probe in the metaphase spread of *L. rubripinis*. Arrowheads indicate B chromosomes. The images were obtained by merging the DAPI-stained patterns (blue) and the signals from the FISH probe (green). Scale bar, 5 µm.
(TIF)

Figure S5 *ihhb* region and primer locations. Red box indicates exon 2 of *ihhb*. The sites that differ between the sequences of *ihhb* paralogs and the *ihhb* ortholog are shown as “T/C” and “C/T” (paralog/ortholog). The primers are shown in Table S5.
(TIF)

Figure S6 Phylogenetic tree of the *ihhb* region sequences (2387 bp) of African cichlids. Tree is constructed by neighbor-joining (NJ), minimum-evolution (ME), and maximum-parsimony (MP) methods. The tree was constructed using the *ihhb* ortholog region of cichlids from three lakes and the *ihhb* paralog region in the B-BAC sequence. *Perissodus microlepis* is used as an outgroup. Bootstrap values by the three methods are indicated at the branch points: top, NJ; middle, ME; bottom, MP. Bootstrap values >49 are indicated.
(TIF)

Figure S7 FISH analysis using DNA probes that include the sequence of DNA markers for LG3 in the metaphase spreads of Lake Victoria cichlids. GM385 (A) and *dmrt4* (B) are DNA markers of LG3 in Tilapia. The images were obtained by merging the DAPI-stained patterns (blue) and the signals from the FISH probe (green). Arrows indicate chromosome 1. The distinct signals indicated by white arrowheads show the GM385 locus and the *dmrt4* region, respectively. Metaphase spreads of *H. chilotes* (A) and *H.* sp. “Matumbi hunter” (B) were used. Scale bar, 5 µm.
(TIF)

Figure S8 A model for how B chromosomes gained their sex determination-related function during evolution. (A) The B chromosome emerged from a sex chromosome (chromosome 1). A sex determination-related gene was also duplicated to appear in the B chromosome. (B) The expression of the gene was suppressed in the heterochromatic state (as indicated by wavy lines). (C) The gene was multiplied within B chromosomes by large-scale duplication events. (D) Some genetic or epigenetic alterations in the B chromosome led to the activation of certain genes, which then gained a function in sex determination.
(TIF)

Table S1 Distribution of the B chromosome in the *L. rubripinnis* pedigree.
(DOC)

Table S2 The size of B chromosomes in wild-caught Lake Victoria cichlid individuals.
(DOC)

Table S3 Known repetitive sequences in the B chromosomes of *H. chilotes*.
(DOC)

Table S4 Protein-coding genes in the B chromosomes.
(DOC)

Table S5 Primer sequences.
(DOC)
