# Supplementary figures and images for: B Chromosomes Have a Functional Effect on Female Sex Determination in Lake Victoria Cichlid Fishes
Source: PLoS Genet. 2011 Aug 18;7(8):e1002203. doi: 10.1371/journal.pgen.1002203 (PMC3158035; doi:10.1371/journal.pgen.1002203)

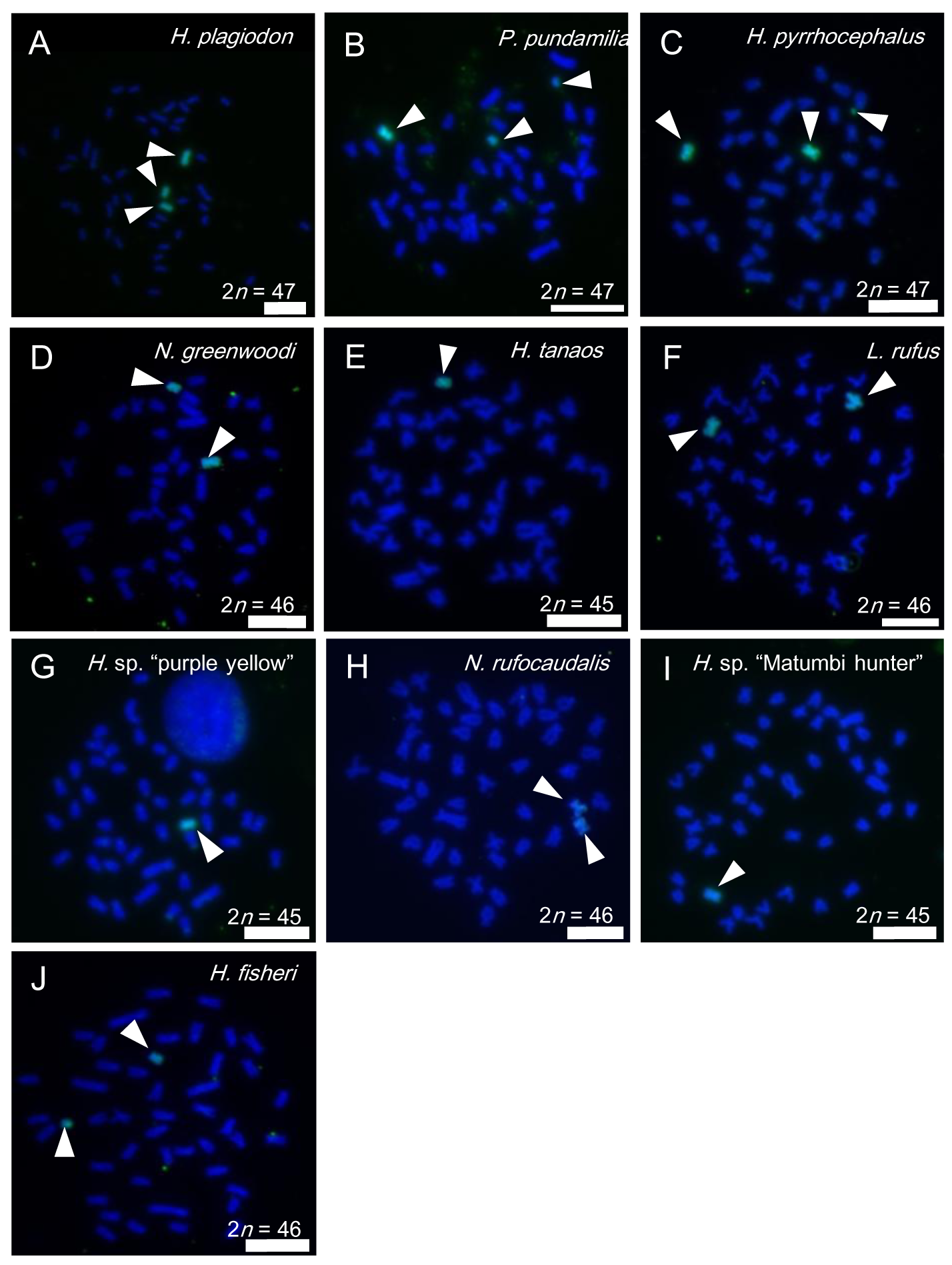

Supplement: Figure S1 — Painting FISH using the B1 probe in the metaphase spread of wild-caught individuals of 10 species of Lake Victoria cichlids. (A) Haplochromis plagiodon, (B) Pundamilia pundamilia, (C) Haplochromis pyrrhocephalus, (D) Neochromis greenwoodi, (E) Haplochromis tanaos, (F) Lithochromis rufus, (G) Haplochromis sp. “purple yellow”, (H) Neochromis rufocaudalis, (I) Haplochromis sp. “Matumbi hunter”, and (J) Haplochromis fisheri. The images were obtained by merging the DAPI-stained patterns (blue) and FISH signals from the probe (green). Arrowheads indicate B chromosomes. Scale bars, 5 µm. (TIF) [file pgen.1002203.s001.tif]

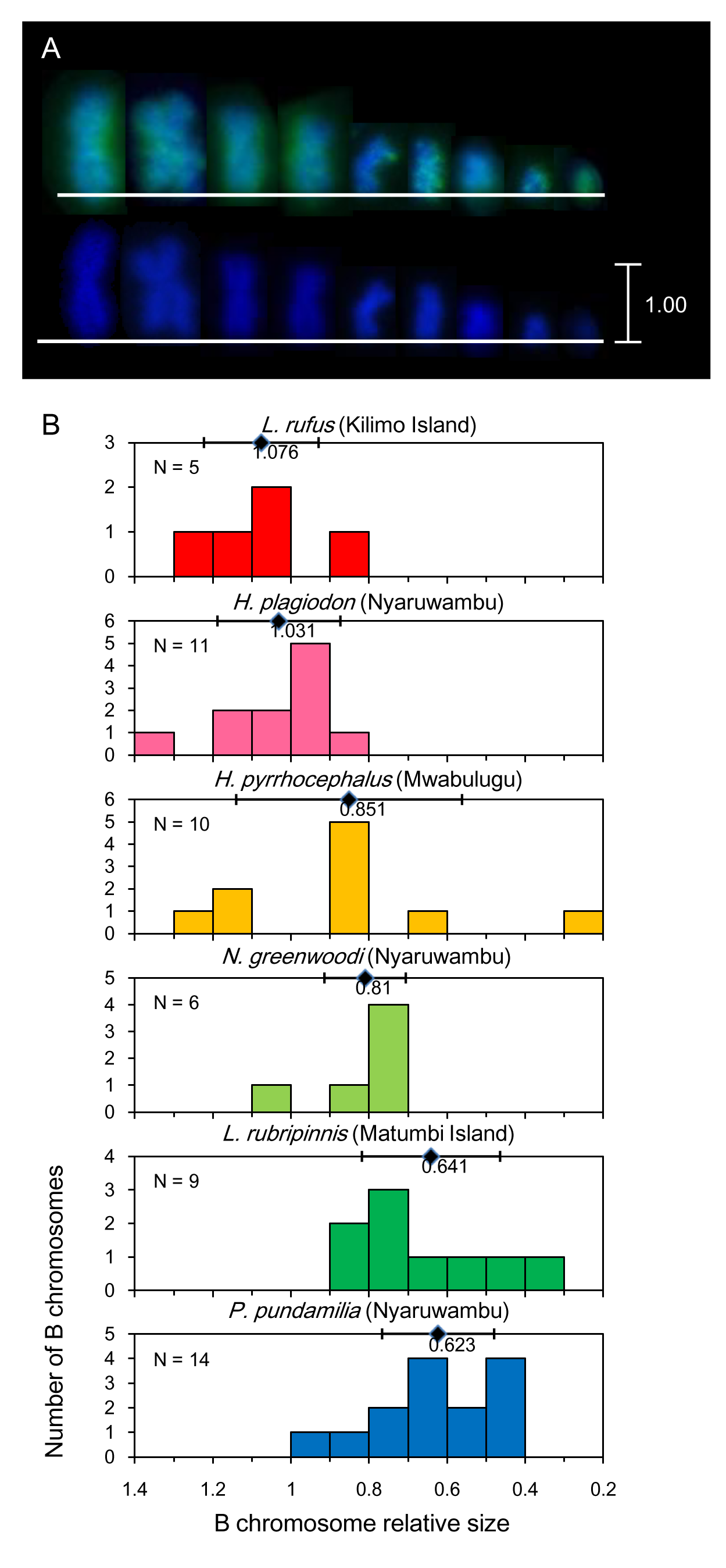

Supplement: Figure S2 — Differences in the size distribution of B chromosomes among populations of Lake Victoria cichlids. (A) Size variation of the B chromosomes painted by the B1 probe (above) and stained with DAPI (bottom). The size of the B chromosomes was calculated by comparison with the mean size of A chromosomes (indicated as 1.00). The images of B chromosomes were adjusted based on the calculated size. B chromosome sizes (species name) of the images are 1.38 (Haplochromis plagiodon), 1.25 (Lithochromis rufus), 1.09 (H. plagiodon), 0.91 (H. plagiodon), 0.79 (L. rubripinnis), 0.70 (L. rubripinnis), 0.51 (Pundamilia pundamilia), 0.40 (L. rubripinnis) and 0.24 (Haplochromis pyrrhocephalus). (B) The differences in the distribution of the relative sizes of B chromosomes. The ratio of the size of a B chromosome to the mean size of the A chromosomes in the same cell was defined as the relative size of the B chromosome. Mean relative size and standard deviation are indicated above each histogram (N = number of B chromosomes). Populations in which more than four B chromosomes were observed were compared. (TIF) [file pgen.1002203.s002.tif]

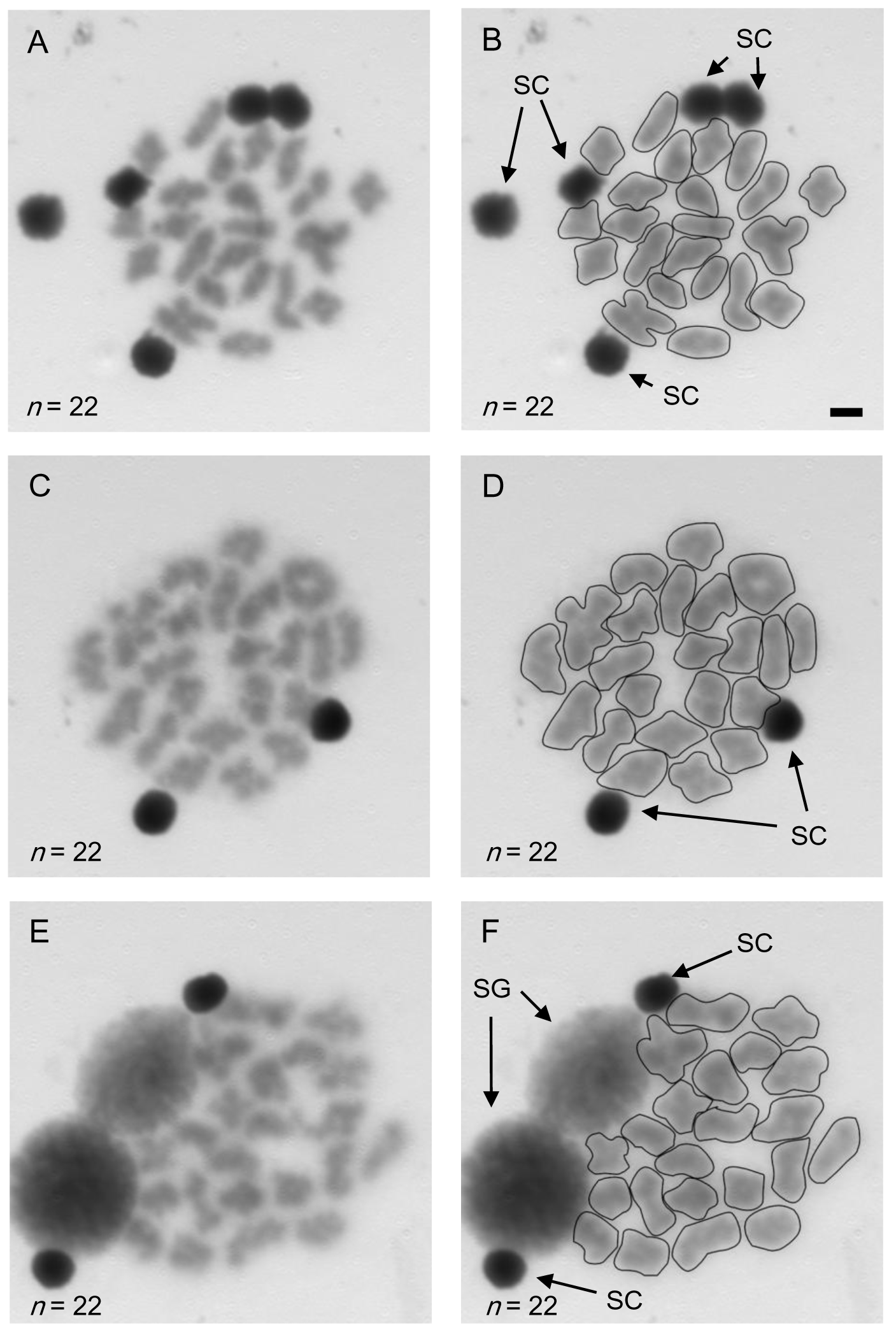

Supplement: Figure S3 — Meiotic chromosomes in the males of the pedigree of L. rubripinnis from the Matumbi island population. Meiotic chromosomes of the three F2 males of L. rubripinnis are indicated in intact (A, C and E) and annotated images (B, D and F). Each chromosome is surrounded by line (B, D and F). The observed haploid number is indicated (n). SC, sperm cells. SG, spermatogonia. Scale bar, 1 µm. (TIF) [file pgen.1002203.s003.tif]

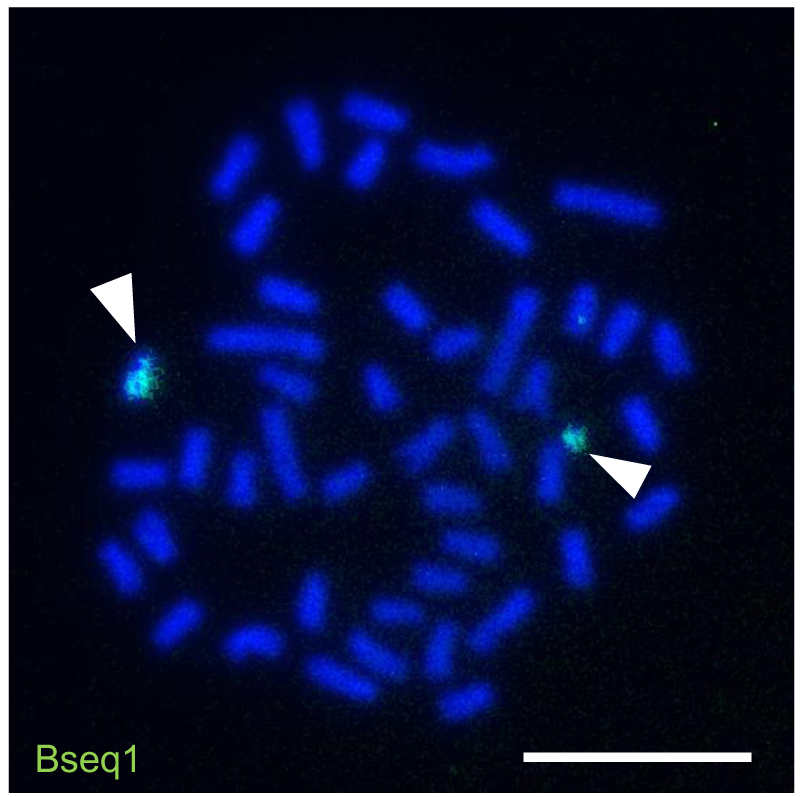

Supplement: Figure S4 — FISH analysis using the Bseq1 probe in the metaphase spread of L. rubripinis. Arrowheads indicate B chromosomes. The images were obtained by merging the DAPI-stained patterns (blue) and the signals from the FISH probe (green). Scale bar, 5 µm. (TIF) [file pgen.1002203.s004.tif]

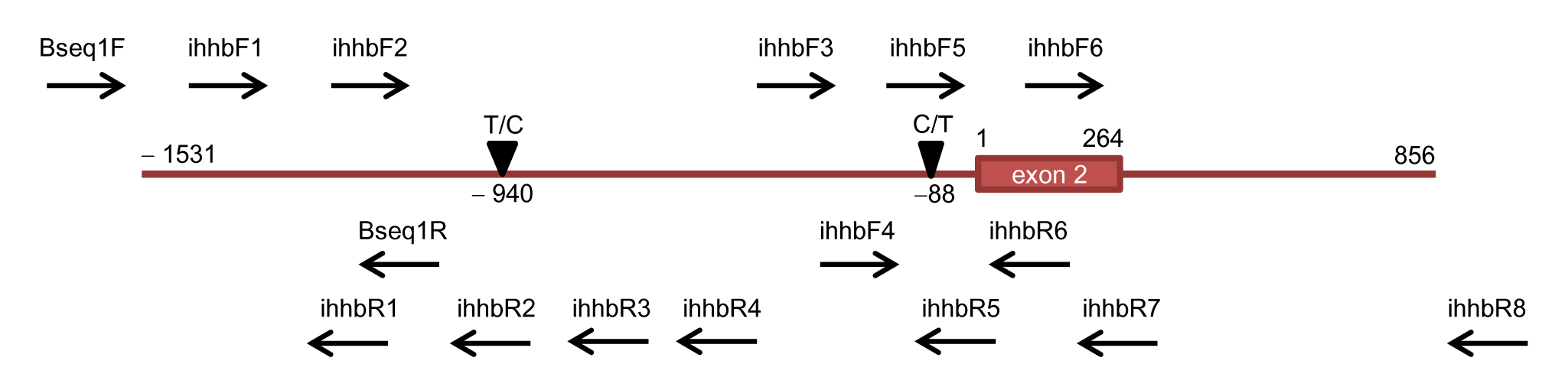

Supplement: Figure S5 — ihhb region and primer locations. Red box indicates exon 2 of ihhb. The sites that differ between the sequences of ihhb paralogs and the ihhb ortholog are shown as “T/C” and “C/T” (paralog/ortholog). The primers are shown in Table S5. (TIF) [file pgen.1002203.s005.tif]

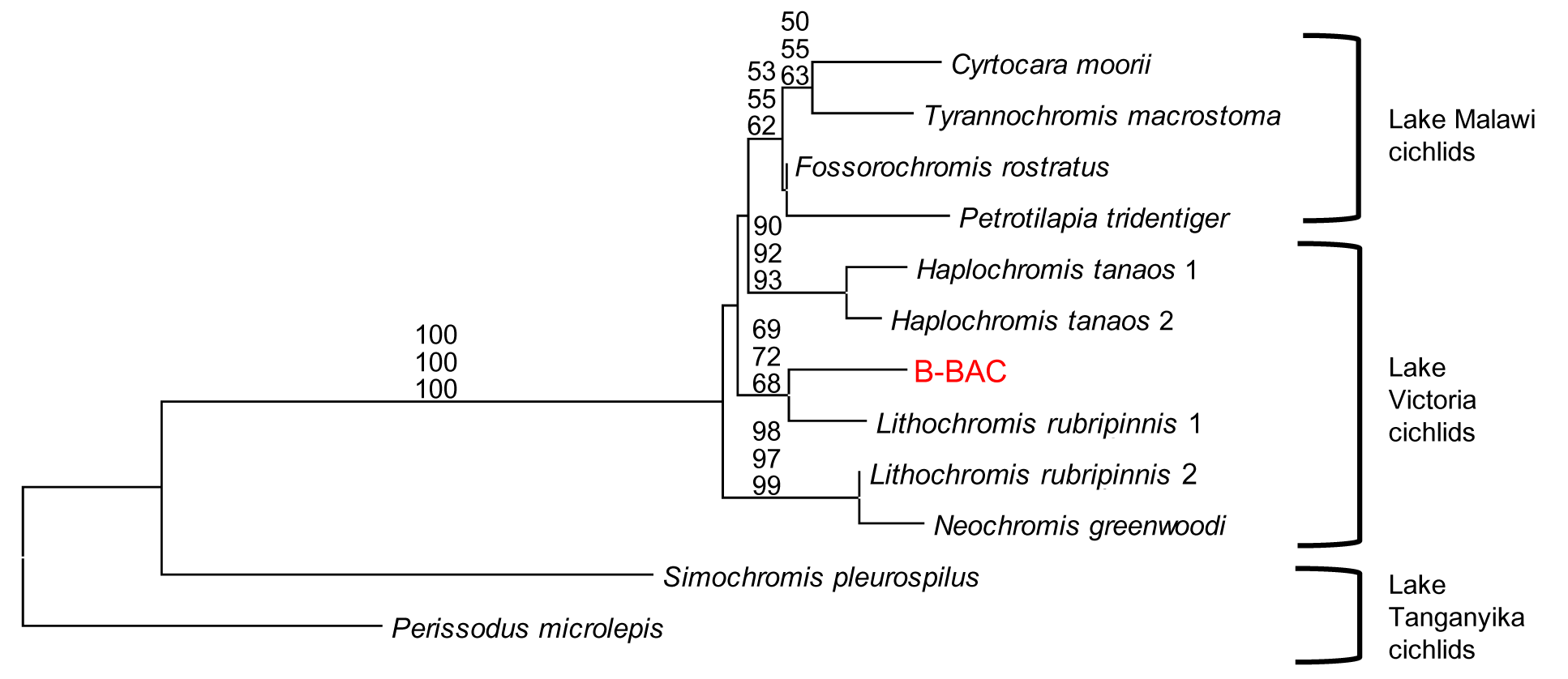

Supplement: Figure S6 — Phylogenetic tree of the ihhb region sequences (2387 bp) of African cichlids. Tree is constructed by neighbor-joining (NJ), minimum-evolution (ME), and maximum-parsimony (MP) methods. The tree was constructed using the ihhb ortholog region of cichlids from three lakes and the ihhb paralog region in the B-BAC sequence. Perissodus microlepis is used as an outgroup. Bootstrap values by the three methods are indicated at the branch points: top, NJ; middle, ME; bottom, MP. Bootstrap values >49 are indicated. (TIF) [file pgen.1002203.s006.tif]

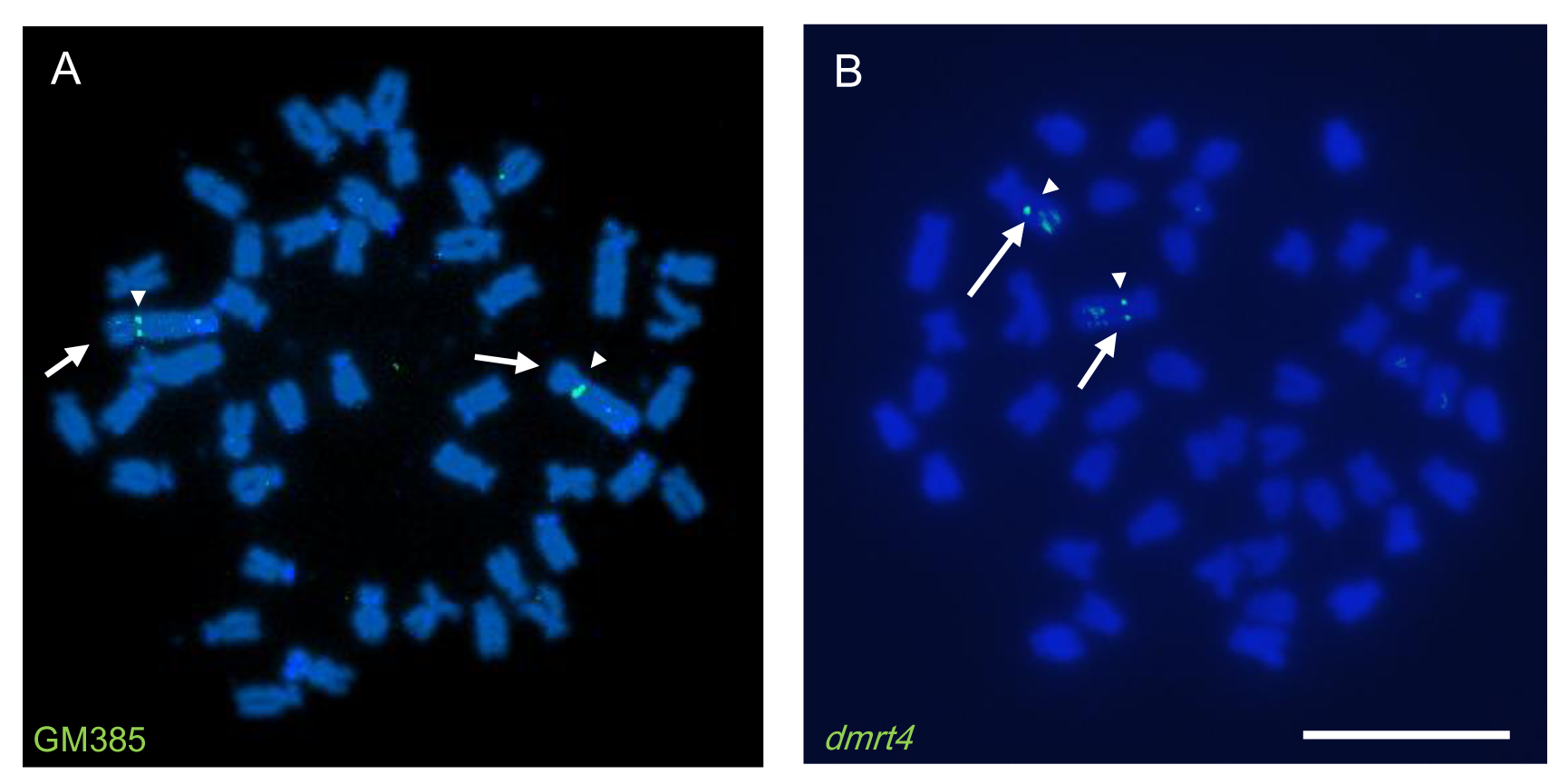

Supplement: Figure S7 — FISH analysis using DNA probes that include the sequence of DNA markers for LG3 in the metaphase spreads of Lake Victoria cichlids. GM385 (A) and dmrt4 (B) are DNA markers of LG3 in Tilapia. The images were obtained by merging the DAPI-stained patterns (blue) and the signals from the FISH probe (green). Arrows indicate chromosome 1. The distinct signals indicated by white arrowheads show the GM385 locus and the dmrt4 region, respectively. Metaphase spreads of H. chilotes (A) and H. sp. “Matumbi hunter” (B) were used. Scale bar, 5 µm. (TIF) [file pgen.1002203.s007.tif]

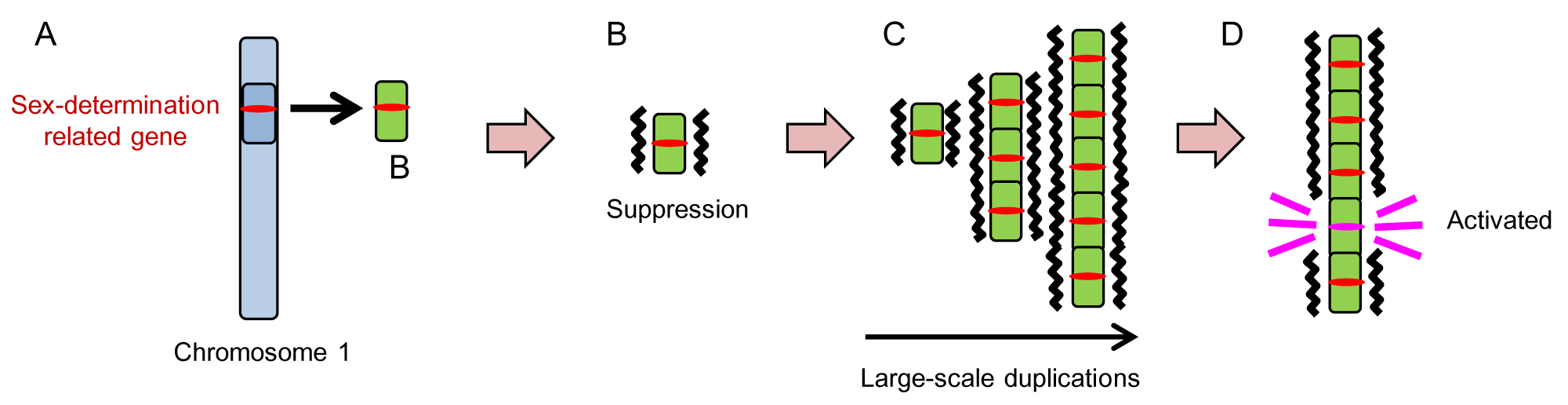

Supplement: Figure S8 — A model for how B chromosomes gained their sex determination-related function during evolution. (A) The B chromosome emerged from a sex chromosome (chromosome 1). A sex determination-related gene was also duplicated to appear in the B chromosome. (B) The expression of the gene was suppressed in the heterochromatic state (as indicated by wavy lines). (C) The gene was multiplied within B chromosomes by large-scale duplication events. (D) Some genetic or epigenetic alterations in the B chromosome led to the activation of certain genes, which then gained a function in sex determination. (TIF) [file pgen.1002203.s008.tif]
