# Supplementary material for: B Chromosomes Have a Functional Effect on Female Sex Determination in Lake Victoria Cichlid Fishes
Source: PLoS Genet. 2011 Aug 18;7(8):e1002203. doi: 10.1371/journal.pgen.1002203 (PMC3158035; doi:10.1371/journal.pgen.1002203)
Supplement: Table S1 — Distribution of the B chromosome in the L. rubripinnis pedigree. (DOC) [file pgen.1002203.s009.doc]

**Table S1. Distribution of the B chromosome in the *L. rubripinnis* pedigree**

| Family | No. | Karyotype | | | | Mean*^a^* | Prevalence*^b^* |
| --- | --- | --- | --- | --- | --- | --- | --- |
|  |  | 44 | 44 + B_1_ | 44 + B_2_ | 44 + B_1_ + B_2_ |  |  |
| F1 family | N = 10 | 5 | 1 | 1 | 3 | 0.80 | 0.50 |
| F2 family | N = 24 | 10 | 3 | 6 | 5 | 0.79 | 0.58 |
| Total | N = 34 | 15 | 4 | 7 | 8 |  |  |

*^a^*The mean number of B chromosomes in a clutch

*^b^*Prevalence rate of individuals that possess a B chromosome(s) in a clutch.
