# Supplementary material for: B Chromosomes Have a Functional Effect on Female Sex Determination in Lake Victoria Cichlid Fishes
Source: PLoS Genet. 2011 Aug 18;7(8):e1002203. doi: 10.1371/journal.pgen.1002203 (PMC3158035; doi:10.1371/journal.pgen.1002203)
Supplement: Table S2 — The size of B chromosomes in wild-caught Lake Victoria cichlid individuals. (DOC) [file pgen.1002203.s010.doc]

**Table S2**. The size of B chromosomes in wild-caught Lake Victoria cichlid individuals

| Species | Locality | Sample No. | Sex | 2*n* | A No. | B No. | B Size (B morphology*^a^*) | | |
| --- | --- | --- | --- | --- | --- | --- | --- | --- | --- |
| *L. rubripinnis* | Matumbi Island | LRBmtb01 | M | 44 | 44 | 0 |  |  |  |
|  |  | LRBmtb02 | M | 44 | 44 | 0 |  |  |  |
|  |  | LRBmtb03 | F | 47 | 44 | 3 | 0.79 (SM) | 0.7 (SM) | 0.4 (M) |
|  |  | LRBmtb04 | F | 47 | 44 | 3 | 0.81 (M) | 0.73 (SM) | 0.57 (SM) |
|  |  | LRBmtb05 | F | 46 | 44 | 2 | 0.81 (ST) | 0.33 (M) |  |
|  |  | LRBmtb06 | F | 45 | 44 | 1 | 0.63 (M) |  |  |
|  | Nyaruwambu | LRBnrw01 | M | 46 | 44 | 2 | 0.95 (M) | 0.85 (M) |  |
|  |  | LRBnrw02 | M | 46 | 44 | 2 | 0.95 (M) | 0.89 (M) |  |
| *H. plagiodon* | Nyaruwambu | HPLnrw01 | M | 46 | 44 | 2 | 1.17 (M) | 0.94 (SM) |  |
|  |  | HPLnrw02 | M | 45 | 44 | 1 | 0.84 (SM) |  |  |
|  |  | HPLnrw03 | M | 45 | 44 | 1 | 0.9 (SM) |  |  |
|  |  | HPLnrw04 | M | 45 | 44 | 1 | 1.14 (SM) |  |  |
|  |  | HPLnrw05 | F | 47 | 44 | 3 | 1.38 (M) | 1.09 (M) | 0.91 (M) |
|  |  | HPLnrw06 | F | 45 | 44 | 1 | 1.06 (M) |  |  |
|  |  | HPLnrw07 | F | 45 | 44 | 1 | 0.99 (M) |  |  |
|  |  | HPLnrw08 | F | 45 | 44 | 1 | 0.92 (SM) |  |  |
| *P. pundamilia* | Nyaruwambu | PPDnrw01 | M | 47 | 44 | 3 | 0.95 (M) | 0.51 (M) | 0.48 (M) |
|  |  | PPDnrw02 | M | 47 | 44 | 3 | 0.81 (M) | 0.64 (M) | 0.47 (M) |
|  |  | PPDnrw03 | M | 46 | 44 | 2 | 0.67 (SM) | 0.6 (M) |  |
|  |  | PPDnrw04 | M | 46 | 44 | 2 | 0.56 (SM) | 0.49 (SM) |  |
|  |  | PPDnrw05 | F | 46 | 44 | 2 | 0.71 (SM) | 0.47 (M) |  |
|  |  | PPDnrw06 | F | 45 | 44 | 1 | 0.62 (SM) |  |  |
|  |  | PPDnrw07 | F | 45 | 44 | 1 | 0.74 (M) |  |  |
| *H. pyrrhocephalus* | Mwabulugu | HPRmwb01 | M | 46 | 44 | 1 | 1.12 (SM) | 0.8 (M) |  |
|  |  | HPRmwb02 | M | 46 | 44 | 2 | 0.84 (M) | 0.8 (M) |  |
|  |  | HPRmwb03 | M | 45 | 44 | 1 | 0.6 (M) |  |  |
|  |  | HPRmwb04 | F | 47 | 44 | 3 | 0.89 (M) | 0.84 (M) | 0.24 (U) |
|  |  | HPRmwb05 | F | 46 | 44 | 1 | 1.25 (M) | 1.13 (M) |  |
|  |  | HPRmwb06 | F | 44 | 44 | 0 |  |  |  |
| *N. greenwoodi* | Nyaruwambu | NGWnrw01 | M | 46 | 44 | 2 | 1.01 (M) | 0.78 (M) |  |
|  |  | NGWnrw02 | M | 45 | 44 | 1 | 0.82 (M) |  |  |
|  |  | NGWnrw03 | M | 44 | 44 | 0 |  |  |  |
|  |  | NGWnrw04 | F | 46 | 44 | 2 | 0.77 (M) | 0.71 (SM) |  |
|  |  | NGWnrw05 | F | 45 | 44 | 1 | 0.77 (M) |  |  |
| *H. tanaos* | Nyegezi Bay | HTNngz01 | M | 45 | 44 | 1 | 0.85 (SM) |  |  |
|  |  | HTNngz02 | M | 44 | 44 | 0 |  |  |  |
|  |  | HTNngz03 | M | 44 | 44 | 0 |  |  |  |
|  |  | HTNngz04 | F | 45 | 44 | 1 | 0.61 (SM) |  |  |
| *L. rufus* | Kilimo Island | LRFklm02 | M | 46 | 44 | 2 | 1.25 (M) | 1.04 (SM) |  |
|  |  | LRFklm03 | M | 46 | 44 | 2 | 1.02 (SM) | 0.88 (M) |  |
|  |  | LRFklm01 | F | 45 | 44 | 1 | 1.19 (M) |  |  |
|  | Matumbi Island | LRFmtb01 | M | 46 | 44 | 2 | 1.08 (SM) | 0.99 (M) |  |
| *H.* sp. "purple yellow" | Nyaruwambu | HPYnrw01 | M | 45 | 44 | 1 | 0.79 (M) |  |  |
|  |  | HPYnrw03 | M | 45 | 44 | 1 | 1.08 (M) |  |  |
|  |  | HPYnrw02 | M | 44 | 44 | 0 |  |  |  |
| *N. rufocaudalis* | Nyegezi Bay | NRFngz01 | M | 45 | 44 | 1 | 1.04 (M) |  |  |
|  |  | NRFngz02 | F | 46 | 44 | 2 | 0.89 (M) | 0.83 (M) |  |
|  | Hippo Island | NRFhpo01 | M | 46 | 44 | 2 | 0.94 (SM) | 0.89 (SM) |  |
| *H.* sp. ”Matumbi hunter” | Nyaruwambu | HMHnrw01 | F | 45 | 44 | 1 | 0.88 (SM) |  |  |
|  |  | HMHnrw02 | F | 45 | 44 | 1 | 0.99 (M) |  |  |
| *H. fisheri* | Nyaruwambu | HFSnrw01 | F | 46 | 44 | 2 | 0.66 (SM) | 0.44 (T) |  |

*^a^*M, metacentric; SM, submetacentric; ST, subtelocentric; T, telocentric; U, unmeasurable.
