# Supplementary material for: B Chromosomes Have a Functional Effect on Female Sex Determination in Lake Victoria Cichlid Fishes
Source: PLoS Genet. 2011 Aug 18;7(8):e1002203. doi: 10.1371/journal.pgen.1002203 (PMC3158035; doi:10.1371/journal.pgen.1002203)
Supplement: Table S3 — Known repetitive sequences in the B chromosomes of H. chilotes. (DOC) [file pgen.1002203.s011.doc]

**Table S3. Known repetitive sequences in the B chromosomes of *H. chilotes***

| Contig  No. | Contig  Length | Contig  From | Contig  To | Subject Name | Class*^a^* | Family | Subject  From | Subject  To | Length | Score | Direction*^b^* |
| --- | --- | --- | --- | --- | --- | --- | --- | --- | --- | --- | --- |
| 1 | 28051 | 1401 | 1465 | RN413 | Tilapia repetitive sequence |  | 309 | 375 | 65 | 228 | d |
|  |  | 1831 | 2093 | BEL32-LTR DR | LTR retrotransposon | Pao | 341 | 606 | 263 | 646 | c |
|  |  | 2302 | 5615 | BEL32-I DR | LTR retrotransposon | Pao | 3552 | 6873 | 3314 | 15481 | c |
|  |  | 6472 | 6644 | piggyBac-N3 DR | DNA transposon | PiggyBac | 1 | 174 | 173 | 366 | c |
|  |  | 11176 | 11251 | CT-rich | Low complexity |  |  |  | 76 | 233 |  |
|  |  | 13513 | 13533 | (TTTA)n | Simple repeat |  |  |  | 21 | 189 |  |
|  |  | 13720 | 13759 | AT rich | Low complexity |  |  |  | 40 | 33 |  |
|  |  | 13947 | 17308 | Expander | LINE | RTE | 1 | 3362 | 3362 | 18543 | d |
|  |  | 19876 | 20001 | RN380 | Tilapia repetitive sequence |  | 707 | 859 | 126 | 342 | d |
|  |  | 20670 | 20801 | RN545 | Tilapia repetitive sequence |  | 14 | 143 | 132 | 959 | c |
|  |  | 20786 | 20889 | RN548 | Tilapia repetitive sequence |  | 395 | 482 | 104 | 308 |  |
|  |  | 20907 | 20994 | RN370 | Tilapia repetitive sequence |  | 778 | 868 | 88 | 588 | c |
|  |  | 21009 | 21708 | Bridge2(Xena) | LINE | Penelope | 395 | 1128 | 700 | 1186 | c |
|  |  | 21713 | 21855 | RN545 | Tilapia repetitive sequence |  | 40 | 144 | 143 | 757 | d |
|  |  | 23053 | 23074 | AT rich | Low complexity |  |  |  | 22 | 22 |  |
|  |  | 24085 | 24131 | RN385 | Tilapia repetitive sequence |  | 474 | 520 | 47 | 261 | d |
|  |  | 25545 | 26993 | Rex1 FurC | LINE | Rex-Babar | 1774 | 3243 | 1449 | 3331 | c |
|  |  | 27261 | 27292 | AT rich | Low complexity |  |  |  | 32 | 25 |  |
| 2 | 13969 | 1 | 137 | RN582 | Tilapia repetitive sequence |  | 73 | 198 | 137 | 319 | d |
|  |  | 262 | 295 | Maui | LINE | L2 | 4659 | 4692 | 34 | 252 | d |
|  |  | 718 | 747 | AT rich | Low complexity |  |  |  | 30 | 30 |  |
|  |  | 1799 | 2058 | Expander | LINE | RTE | 937 | 1203 | 260 | 729 | c |
|  |  | 2214 | 2403 | Expander | LINE | RTE | 396 | 594 | 190 | 422 | c |
|  |  | 3821 | 3859 | AT rich | Low complexity |  |  |  | 39 | 25 |  |
|  |  | 4115 | 4159 | AT rich | Low complexity |  |  |  | 45 | 24 |  |
|  |  | 4825 | 4942 | RN428 | Tilapia repetitive sequence |  | 660 | 772 | 118 | 352 | d |
|  |  | 4987 | 5016 | AT rich | Low complexity |  |  |  | 30 | 30 |  |
|  |  | 5385 | 5632 | RN359 | Tilapia repetitive sequence |  | 593 | 844 | 248 | 1055 | c |
|  |  | 5943 | 5973 | TC1DR3 | DNA transposon | TcMar-Tc1 | 61 | 91 | 31 | 277 | d |
|  |  | 5991 | 6021 | AT rich | Low complexity |  |  |  | 31 | 24 |  |
|  |  | 6305 | 6640 | RN449 | Tilapia repetitive sequence |  | 174 | 541 | 336 | 669 | d |
|  |  | 8176 | 8331 | HATN7 DR | DNA transposon | hAT | 1 | 167 | 156 | 43 | d |
|  |  | 9327 | 9417 | RN379 | Tilapia repetitive sequence |  | 512 | 615 | 91 | 420 | c |
|  |  | 10614 | 10640 | RN444 | Tilapia repetitive sequence |  | 927 | 953 | 27 | 228 | d |
|  |  | 10714 | 10792 | DNA-X-7 DR | DNA transposon |  | 565 | 642 | 79 | 297 | c |
|  |  | 10806 | 11046 | TDR8C | DNA transposon |  | 690 | 921 | 241 | 449 | c |
|  |  | 11048 | 11142 | RN413 | Tilapia repetitive sequence |  | 117 | 208 | 95 | 628 | d |
|  |  | 11112 | 11234 | RN518 | Tilapia repetitive sequence |  | 159 | 281 | 123 | 672 | d |
|  |  | 11541 | 11586 | AT rich | Low complexity |  |  |  | 46 | 39 |  |
|  |  | 11960 | 12045 | hAT-N58 DR | DNA transposon | hAT-Ac | 721 | 808 | 86 | 352 | d |
| 3 | 12545 | 34 | 67 | Maui | LINE | L2 | 4659 | 4692 | 34 | 252 | c |
|  |  | 192 | 355 | RN582 | Tilapia repetitive sequence |  | 73 | 225 | 164 | 478 | d |
|  |  | 1756 | 2072 | SINE AFC | SINE |  | 5 | 339 | 317 | 1839 | c |
|  |  | 2249 | 2340 | hAT-N49 DR | DNA transposon | hAT-Ac | 1021 | 1111 | 92 | 348 | c |
|  |  | 3632 | 3689 | Gypsy151-I DR | LTR retrotransposon | Gypsy | 338 | 392 | 58 | 229 | d |
|  |  | 4240 | 4371 | Gypsy151-I DR | LTR retrotransposon | Gypsy | 994 | 1125 | 132 | 258 | d |
|  |  | 6261 | 9130 | Gypsy149-I DR | LTR retrotransposon | Gypsy | 2724 | 5581 | 2870 | 2985 | d |
|  |  | 10721 | 10999 | BEL32-LTR DR | LTR retrotransposon | Pao | 341 | 606 | 279 | 593 | c |
|  |  | 11657 | 12135 | RN449 | Tilapia repetitive sequence |  | 39 | 541 | 479 | 1025 | d |
|  |  | 12294 | 12460 | Maui | LINE | L2 | 4661 | 4797 | 167 | 338 | d |
| 4 | 8553 | 167 | 400 | RN385 | Tilapia repetitive sequence |  | 483 | 697 | 234 | 555 | d |
|  |  | 1834 | 1864 | RN82 | Tilapia repetitive sequence |  | 603 | 633 | 31 | 247 | c |
|  |  | 1869 | 2091 | SINE AFC | SINE |  | 13 | 250 | 223 | 1478 | c |
|  |  | 2093 | 2378 | RN539 | Tilapia repetitive sequence |  | 131 | 415 | 286 | 2435 | d |
|  |  | 3474 | 3779 | SINE AFC | SINE |  | 4 | 333 | 306 | 2255 | d |
|  |  | 4147 | 4252 | Expander | LINE |  | 3043 | 3150 | 106 | 339 | c |
|  |  | 5113 | 5174 | AT rich | Low complexity |  |  |  | 62 | 34 |  |
|  |  | 5720 | 5761 | AT rich | Low complexity |  |  |  | 42 | 28 |  |
|  |  | 6964 | 7132 | RN596 | Tilapia repetitive sequence |  | 1 | 175 | 169 | 985 | d |
|  |  | 7242 | 7312 | RN572 | Tilapia repetitive sequence |  | 319 | 389 | 71 | 448 | c |
|  |  | 7313 | 7405 | TC2 FR4 | DNA transposon | TcMar-Tc2 | 252 | 364 | 93 | 469 | c |
|  |  | 7406 | 7975 | RN400 | Tilapia repetitive sequence |  | 29 | 607 | 570 | 4310 | d |
|  |  | 8147 | 8259 | (TTAA)n | Simple repeat |  |  |  | 113 | 340 |  |
|  |  | 8267 | 8370 | (TTTA)n | Simple repeat |  |  |  | 104 | 181 |  |
| 5 | 6389 | 9 | 267 | BEL32-LTR DR | LTR retrotransposon | Pao | 371 | 606 | 259 | 592 | d |
|  |  | 425 | 6389 | BEL32-I DR | LTR retrotransposon | Pao | 861 | 6867 | 5965 | 26262 | d |
| 6 | 5380 | 111 | 153 | (TG)n | Simple repeat |  |  |  | 43 | 210 |  |
|  |  | 4437 | 4481 | RN453 | Tilapia repetitive sequence |  | 363 | 414 | 45 | 259 | d |
|  |  | 4537 | 4560 | AT rich | Low complexity |  |  |  | 24 | 24 |  |
|  |  | 4588 | 4694 | RN453 | Tilapia repetitive sequence |  | 556 | 666 | 107 | 516 | d |
|  |  | 4856 | 5071 | SINE FR2 | SINE | V | 180 | 388 | 216 | 818 | d |
| 7 | 4224 | 3138 | 3174 | AT rich | Low complexity |  |  |  | 37 | 23 |  |
|  |  | 3988 | 4062 | SINE FR2 | SINE | V | 257 | 341 | 75 | 252 | c |
| 8 | 3995 | 1 | 303 | RN449 | Tilapia repetitive sequence |  | 39 | 350 | 303 | 587 | c |
|  |  | 963 | 1047 | (CAGGA)n | Simple repeat |  |  |  | 85 | 211 |  |
|  |  | 1658 | 1726 | RN170 | Tilapia repetitive sequence |  | 964 | 1032 | 69 | 340 | c |
|  |  | 2089 | 2534 | RN449 | Tilapia repetitive sequence |  | 69 | 541 | 446 | 879 | c |
|  |  | 3095 | 3323 | SINE AFC | SINE |  | 1 | 224 | 229 | 1099 | c |
| 9 | 3638 | 1 | 165 | HarbingerN3 DR | DNA transposon | Harbinger | 900 | 1064 | 165 | 700 | d |
|  |  | 238 | 493 | SINE AFC | SINE |  | 7 | 324 | 256 | 1480 | d |
|  |  | 515 | 625 | RN549 | Tilapia repetitive sequence |  | 586 | 686 | 111 | 288 | c |
|  |  | 1582 | 1634 | AT rich | Low complexity |  |  |  | 53 | 25 |  |
|  |  | 2945 | 3045 | CR1-41 DR | LINE | L2 | 1396 | 1496 | 101 | 236 | d |
| 10 | 3251 | 195 | 734 | CR1-27 DR | LINE | L2 | 1025 | 1572 | 540 | 753 | d |
|  |  | 780 | 902 | (TATG)n | Simple repeat |  |  |  | 123 | 261 |  |
|  |  | 971 | 1044 | CR1-27 DR | LINE | L2 | 1788 | 1865 | 74 | 248 | d |
|  |  | 1143 | 1164 | (CAAT)n | Simple repeat |  |  |  | 22 | 198 |  |
|  |  | 2371 | 2528 | ERV3 DR-I | LTR retrotransposon | ERV1 | 4575 | 4847 | 158 | 324 | c |
| 11 | 2831 | 1569 | 1650 | HE1 DR1 | SINE | V | 18 | 112 | 82 | 307 | d |
|  |  | 1644 | 1855 | SINE FR2 | SINE | V | 153 | 373 | 212 | 574 | d |
| 12 | 2573 | 264 | 320 | RN324 | Tilapia repetitive sequence |  | 1 | 58 | 57 | 462 | d |
|  |  | 322 | 346 | (A)n | Simple repeat |  |  |  | 25 |  |  |
|  |  | 455 | 689 | RN592 | Tilapia repetitive sequence |  | 1 | 220 | 235 | 1438 | d |
|  |  | 574 | 595 | AT rich | Low complexity |  |  |  | 22 |  |  |
|  |  | 660 | 840 | RN315 | Tilapia repetitive sequence |  | 1 | 201 | 181 | 1033 | d |
|  |  | 851 | 935 | DNA-8-15 DR | DNA transposon | hAT-AC | 1 | 90 | 85 | 284 | c |
| 13 | 2552 | 737 | 773 | AT rich | Low complexity |  |  |  | 37 | 23 |  |
|  |  | 1585 | 1711 | Maui | LINE | L2 | 4572 | 4695 | 127 | 266 | c |
|  |  | 1943 | 1965 | (CAATT)n | Simple repeat |  |  |  | 23 | 207 |  |
|  |  | 1966 | 2220 | Maui | LINE | L2 | 4546 | 4771 | 255 | 588 | c |
|  |  | 2221 | 2376 | RN170 | Tilapia repetitive sequence |  | 496 | 651 | 156 | 987 | d |
|  |  | 2314 | 2348 | Maui | LINE | L2 | 4262 | 4296 | 35 | 239 | c |
|  |  | 2377 | 2551 | Maui | LINE | L2 | 4009 | 4183 | 175 | 914 | c |
| 14 | 2146 | 3 | 237 | RN46 | Tilapia repetitive sequence |  | 3 | 236 | 235 | 1427 | d |
|  |  | 175 | 209 | Maui | LINE | L2 | 4262 | 4296 | 35 | 239 | c |
|  |  | 238 | 1366 | Maui | LINE | L2 | 3058 | 4183 | 1129 | 4869 | c |
| 15 | 1576 | 83 | 820 | RN441 | Tilapia repetitive sequence |  | 1 | 732 | 738 | 6670 | d |
|  |  | 1135 | 1541 | CR1-12 DR | LINE | L2 | 928 | 1341 | 407 | 513 | d |
| 16 | 1169 | 552 | 703 | RN413 | Tilapia repetitive sequence |  | 248 | 419 | 152 | 363 | d |
|  |  | 925 | 1100 | (TAAA)n | Simple repeat |  |  |  | 176 | 185 |  |
|  |  | 1126 | 1169 | AT rich | Low complexity |  |  |  | 44 | 23 |  |
| 17 | 939 |  |  | No hit |  |  |  |  |  |  |  |
| 18 | 674 |  |  | No hit |  |  |  |  |  |  |  |

*^a^*LTR, long terminal repeat; LINE, long interspersed nuclear element; SINE, short interspersed nuclear element.

*^b^*Direction of the repetitive sequence: “d” and “c” indicate direct and complementary strand, respectively.
