# Supplementary material for: B Chromosomes Have a Functional Effect on Female Sex Determination in Lake Victoria Cichlid Fishes
Source: PLoS Genet. 2011 Aug 18;7(8):e1002203. doi: 10.1371/journal.pgen.1002203 (PMC3158035; doi:10.1371/journal.pgen.1002203)
Supplement: Table S4 — Protein-coding genes in the B chromosomes. (DOC) [file pgen.1002203.s012.doc]

**Table S4**. Protein-coding genes in the B chromosomes

| Contig  No. | Contig  Length | Contig  From | Contig  To | Gene  Accession No. | Gene Name | Species | Gene  From | Gene  To | Coding Region | Length (a.a.) | Similarity |
| --- | --- | --- | --- | --- | --- | --- | --- | --- | --- | --- | --- |
| 1 | 28051 | 7353 | 7616 | NP_571163.1 | Indian hedgehog homolog b | *Danio rerio* | 101 | 187 | exon 2 | 87 | 94.3% |
|  |  | 20091 | 20192 | NP_001014354.1 | lysosomal α-mannosidase | *Danio rerio* | 420 | 453 | exon 10 | 34 | 88.2% |
|  |  | 20361 | 20468 |  |  |  | 455 | 490 | exon 11 | 36 | 75.0% |
|  |  | 22176 | 22286 |  |  |  | 491 | 527 | exon 12 | 37 | 75.7% |
|  |  | 22322 | 22417 |  |  |  | 633 | 662 | exon 15 | 32 | 78.1% |
|  |  | 22628 | 22732 |  |  |  | 668 | 702 | exon 15 | 35 | 82.9% |
|  |  | 23182 | 23280 |  |  |  | 705 | 737 | exon 16 | 33 | 81.8% |
|  |  | 23476 | 23562 |  |  |  | 739 | 767 | exon 17 | 29 | 86.2% |
|  |  | 23673 | 23753 |  |  |  | 768 | 794 | exon 18 | 27 | 77.8% |
|  |  | 23859 | 24083 |  |  |  | 795 | 868 | exon 19 | 75 | 69.3% |
|  |  | 24700 | 24849 |  |  |  | 869 | 918 | exon 20 | 50 | 84.0% |
|  |  | 24983 | 25072 |  |  |  | 919 | 947 | exon 21 | 30 | 63.3% |
|  |  | 27659 | 27745 |  |  |  | 951 | 981 | exon 22 | 29 | 72.4% |
| 2 | 13969 | 4341 | 4760 | NP_001093575 | ribonuclease-like 2 | *Danio rerio* | 1 | 140 | Whole | 140 | 40.8% |
| 3 | 12545 | 3952 | 4239 | XP_002662899.1 | VPS10 domain receptor protein SORCS 3–like | *Danio rerio* | 68 | 160 | exon 1 | 96 | 32.3% |
|  |  | 5372 | 6253 |  |  |  | 333 | 621 | exon 2 | 294 | 30.5% |
| 6 | 5380 | 917 | 994 | CAG10072.1 | ryanodine receptor–like unnamed protein | *Tetraodon nigroviridis* | 3090 | 3115 | exon 60 | 26 | 80.8% |
|  |  | 1308 | 1424 |  |  |  | 3117 | 3159 | exon 61 | 39 | 78.9% |
|  |  | 1523 | 1645 |  |  |  | 3162 | 3203 | exon 62 | 41 | 69.0% |
|  |  | 2690 | 2869 |  |  |  | 3207 | 3266 | exon 62 | 60 | 69.8% |
|  |  | 2992 | 3204 |  |  |  | 3274 | 3344 | exon 63 | 71 | 71.8% |
|  |  | 3582 | 3668 |  |  |  | 3347 | 3375 | exon 64 | 29 | 75.9% |
|  |  | 3780 | 3857 |  |  |  | 3380 | 3401 | exon 65 | 26 | 76.9% |
|  |  | 4049 | 4177 |  |  |  | 3419 | 3458 | exon 66 | 43 | 62.8% |
|  |  | 4298 | 4360 |  |  |  | 3459 | 3479 | exon 67 | 21 | 52.4% |
| 12 | 2573 | 1752 | 1853 | CAG10072.1 | ryanodine receptor–like unnamed protein | *Tetraodon nigroviridis* | 3561 | 3594 | exon 70 | 34 | 73.5% |
| 8 | 3995 | 2577 | 2651 | CAG10072.1 | ryanodine receptor–like unnamed protein | *Tetraodon nigroviridis* | 3783 | 3807 | exon 79 | 25 | 96.0% |
|  |  | 2864 | 2950 |  |  |  | 3809 | 3837 | exon 80 | 29 | 89.7% |
|  |  | 3432 | 3557 |  |  |  | 3839 | 3880 | exon 81 | 42 | 83.3% |
|  |  | 3906 | 3995 |  |  |  | 3881 | 3939 | exon 82 | 30 | 86.7% |
