# Supplementary material for: B Chromosomes Have a Functional Effect on Female Sex Determination in Lake Victoria Cichlid Fishes
Source: PLoS Genet. 2011 Aug 18;7(8):e1002203. doi: 10.1371/journal.pgen.1002203 (PMC3158035; doi:10.1371/journal.pgen.1002203)
Supplement: Table S5 — Primer sequences. (DOC) [file pgen.1002203.s013.doc]

**Table S5. Primer sequences**

| Primer name | Region | 5' to 3' sequence |
| --- | --- | --- |
| ihhbF1 | *ihhb* | GCACCTTTCACTTCACGTGT |
| ihhbF2 | *ihhb* | GACATCTGGAATTGGTTTACGC |
| ihhbF3 | *ihhb* | ATAGCCTCCCTCCTCCGTTA |
| ihhbF4 | *ihhb* | AAAGGGGGTTTGGTGAAGTC |
| ihhbF5 | *ihhb* | TATTGATCCAGACACGTCCTGT |
| ihhbF6 | *ihhb* | GATTCGACTGGGTCTACTACGAG |
| ihhbR1 | *ihhb* | TAGTCTGAAGAGTTGGTGCGTT |
| ihhbR2 | *ihhb* | ATCTAGACAACCCAAAATGTGA |
| ihhbR3 | *ihhb* | CATTAACCTCCTAACACCCGAAC |
| ihhbR4 | *ihhb* | AGTCACGGCGTCTTGGATAAC |
| ihhbR5 | *ihhb* | ACTGAAGCCTCGTCCTCTGC |
| ihhbR6 | *ihhb* | GCCAGAGAGTTCAGCCTGTC |
| ihhbR7 | *ihhb* | GTAGTAGACCCAGTCGAATCCA |
| ihhbR8 | *ihhb* | CACTGCCTCTCAATTGTTTATCT |
| Bseq1F | Bseq1 & *ihhb* | ATGAAGAGGGAAACTAAAGCTGT |
| Bseq1R | Bseq1 & *ihhb* | CCAATTCATAGACGGGTTTCAG |
